# Novel Antimicrobial Protein Fibroblast Growth Factor 8 Accelerates Skin Wound Healing via Directly Inhibiting Bacteria and Activating Glycolysis

**DOI:** 10.1002/advs.202500388

**Published:** 2025-06-30

**Authors:** Ya‐Zhen Hu, Ting Wang, Chang‐Song Wu, Jie Wang, Xue‐Qing Han, Yong‐An Zhang, Xu‐Jie Zhang

**Affiliations:** ^1^ National Key Laboratory of Agricultural Microbiology Hubei Hongshan Laboratory Engineering Research Center of Green Development for Conventional Aquatic Biological Industry in the Yangtze River Economic Belt Ministry of Education College of Fisheries Huazhong Agricultural University Wuhan 430070 China; ^2^ School of Marine Sciences Ningbo University Ningbo 315832 China; ^3^ Shenzhen Institute of Nutrition and Health Huazhong Agricultural University Wuhan 430070 China; ^4^ Shenzhen Branch, Guangdong Laboratory for Lingnan Modern Agriculture, Genome Analysis Laboratory of the Ministry of Agriculture, Agricultural Genomics Institute at Shenzhen Chinese Academy of Agricultural Sciences Shenzhen 518000 China

**Keywords:** antimicrobial protein, FGF8, glycolysis, wound healing

## Abstract

Repairing and preventing infections of skin wounds are crucial for the body health. Fibroblast growth factors (FGFs) have functions of repairing skin wounds, while antimicrobial peptides/proteins (AMPs) can prevent the infections of skin. Therefore, if FGFs can act like AMPs, they will have better application potential in promoting skin wound healing. Here, it is found that grass carp fibroblast growth factor 8a (gcFGF8a) has broad‐spectrum and potent antibacterial activity through disrupting the cell membrane, indicating that FGF8 is an uncovered AMP. Further study revealed that gcFGF8a can accelerate skin wound healing through dual functions: inhibiting bacterial and activating of glycolysis. Mechanistically, gcFGF8a/FGFR4 signaling induces epithelial cell glycolytic programs for barrier remodeling through the mTORC1/HIF1α pathway. Interestingly, it is found that mouse FGF8b (mFGF8b), the homolog of gcFGF8a, also has antibacterial and skin wound repair activities, indicating that FGF8 is a functionally conserved molecule in vertebrates. To the knowledge, FGF8 is the only member of the FGF family with both antibacterial and wound repair functions at present, greatly enriching the understanding of this pivotal molecule. Furthermore, these findings highlighted the potential application of FGF8 as a skin wound repair drug.

## Graphical abstract

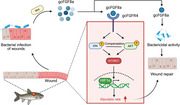

## 1 Introduction

Wound healing is a complex process involving hemostasis, inflammation, re‐epithelialization, and remodeling.^[^ ^1^ ^]^ However, the progression of the healing process may face setbacks or hindrances due to various internal factors or the occurrence of wound infections.^[^ ^2^, ^3^, ^4^, ^5^ ^]^ Over the past decade, antimicrobial peptides/proteins (AMPs) have gained considerable attention as potential therapeutic candidates for treatment of wound infections due to their diverse functions in microbial killing, inflammation, and wound healing.^[^ ^6^ ^]^ Importantly, AMPs are considered to have broad‐spectrum antibacterial activity, low toxicity, and the lowest resistance compared to antibiotics.^[^ ^7^ ^]^ Among them, defensins (such as α‐defensins and β‐defensins), LL‐37, and histatins (histatins‐1 and histatins‐5) have been reported to possess both antibacterial and tissue regenerative properties.^[^ ^8^ ^]^ For instance, LL‐37 stimulates cytokine and chemokine production and promotes epithelial cell migration and angiogenesis.^[^ ^9^, ^10^ ^]^ Overall, AMPs have shown great promise in promoting wound healing and preventing infections. However, the specific mechanism of the interaction between AMP and wound repair are largely unknown.

Apart from infection, another critical microenvironmental condition during the wound repair process is the limited oxygen supply.^[^ ^11^, ^12^ ^]^ Initial tissue damage triggers vascular injury, leading to acute tissue hypoxia. And the increased oxygen consumption by infiltrating inflammatory and stromal cells further decreases tissue oxygenation, resulting in prolonged chronic hypoxia.^[^ ^13^ ^]^ Hypoxia‐inducible factor‐1 α (HIF‐1α), a key transcription factor under hypoxic conditions, plays a crucial role in various aspects of wound healing, including cell energy metabolism, angiogenesis, cell proliferation, and cell migration.^[^ ^14^, ^15^ ^]^ For instance, HIF‐1α facilitates the delivery of oxygen and nutrients to the wound site and stimulates angiogenesis by binding to hypoxia response elements.^[^ ^16^, ^17^ ^]^ In cutaneous wounds, HIF‐1α also mediates the inflammatory response by regulating glycolytic metabolism.^[^ ^18^, ^19^ ^]^ These preliminary studies underscore the spatiotemporal activation and regulation of HIF‐1 signaling in the wound healing process. However, the crosstalk between microenvironmental signals such as AMP‐induced signals and HIF‐1α signaling pathway in tissue repair processes remains to be explored.

Fibroblast growth factors (FGFs), originally named for their role in promoting fibroblast proliferation, constitute a family of 23 members that orchestrate various cellular processes including migration, proliferation, differentiation, survival, metabolic activity, and neural function across diverse cell types.^[^ ^20^, ^21^ ^]^ FGF8, a member of the FGF family, has conserved structure and diverse biological activities.^[^ ^22^ ^]^ It interacts with FGF receptors (FGFRs) to orchestrates the growth, differentiation, and organogenesis of various cell types during embryonic development.^[^ ^23^ ^]^ Furthermore, FGF8 promotes the migration and proliferation of neural crest cells, which give rise to various cell types in the neural crest, including adipocytes, chondrocytes, and neuronal cells.^[^ ^24^ ^]^ Functional studies demonstrated that gcFGF8a expression is significantly upregulated in response to bacterial infections. Subsequent biochemical analysis revealed that gcFGF8a exhibits a “cationic, amphiphilic” structure ‐ a hallmark of classical AMPs.^[^ ^25^ ^]^ These characteristics strongly suggest that FGF8a may be involved in antibacterial immune defense. Our results showed that FGF8 has broad‐spectrum and potent antibacterial activity, uncovering FGF8 as a novel AMP in vertebrates, extends its biological role beyond traditional developmental functions. Moreover, FGF8 can speed up skin wound healing via directly inhibiting bacteria and activating glycolysis through the mTOR‐HIF1α pathway. Our findings underscore FGF8's evolutionary conservation in antibacterial and wound repair functions across species, laying important foundation for the application of FGF8 as a wound repair drug.

## 2 Results

### 2.1 gcFGF8a is a New Participant in Grass Carp Defense Against *A. hydrophila* Infection

**Figure 1:**
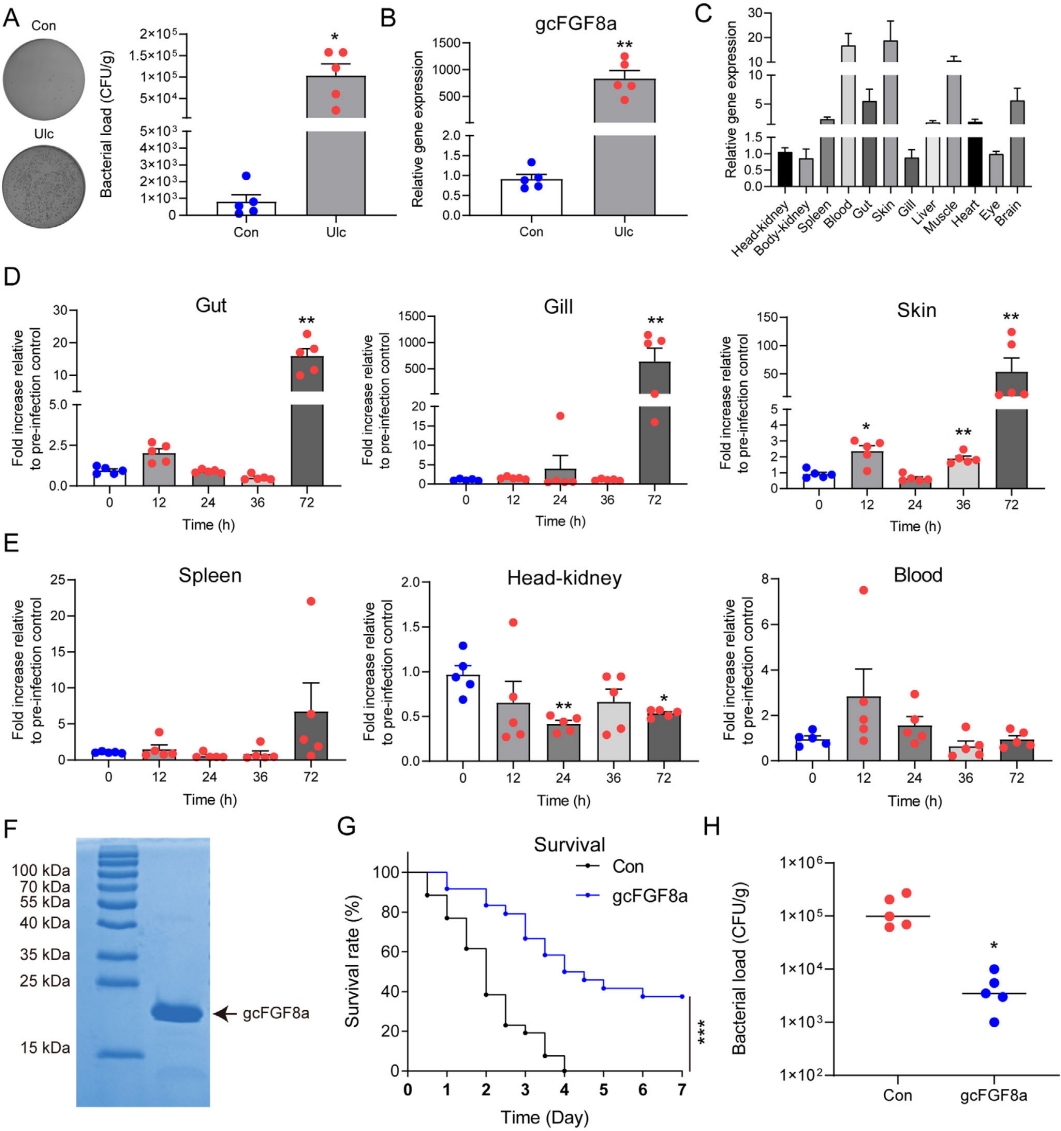
gcFGF8a plays an antibacterial role against *A. hydrophila* infection in grass carp. A) Bacterial load at the ulcer area. Tissues from naturally ulcerated areas (Ulc) were homogenized, serially diluted, and cultured onto TSA plates to quantify bacterial loads, with healthy tissues as a control (Con), *n* = 5. B) Expression levels of *gcFGF8a* at the ulcer area. The fold changes in *gcFGF8a* expression between naturally ulcerated and healthy fish tissues were quantified by RT‐qPCR, with the *18S rRNA* gene as internal control, *n* = 5. C) Expression profiles of *gcFGF8a* in healthy grass carp. *gcFGF8a* expression levels were analyzed by RT‐qPCR, with *18S rRNA* gene as internal control, *n* = 12. D,E) Expression profiles of *gcFGF8a* after *A. hydrophila* infection. The fold changes of *gcFGF8a* expression after *A. hydrophila* infection in mucosal immune tissues (D) and systemic immune tissues (E) were calculated by comparing the infected group with the pre‐infection control by RT‐qPCR, *n* = 5. F) SDS‐PAGE analysis of the recombinant gcFGF8a purified from *E. coli*. G) Survival rate after gcFGF8a application. The treatment with gcFGF8a (1 µg g^−1^) injected intraperitoneally started at 12 h post‐infection (*A. hydrophila*, 2 × 10^6^ CFU/fish), and the survival rate was recorded every 12 h for 7 days, *n* = 30. H) Bacterial load quantification after application of gcFGF8a or PBS (Con) in vivo following *A. hydrophila* infection. Skin extracted 3 days after infection was used for CFU counting to determine bacterial load, *n* = 5. *p ≤ 0.05, **p ≤ 0.01, ***p ≤ 0.001.

In mammals, *FGF8* forms different splice variants through alternative splicing.^[^ ^26^ ^]^ However, in teleosts, *FGF8* does not have splice variants but consists of two isoforms (*FGF8a* and *FGF8b*), with *FGF8a* being the ortholog of tetrapod *FGF8* (Figure S1, Supporting Information). Additionally, the structure and sequence of FGF8 exhibits a high degree of conservation during evolution (Figure S2A,B, Supporting Information). Using grass carp as a model, in the ulcerated fish, we found that the bacterial load and *gcFGF8a* expression increased in the ulcer area (**Figure** **1**A,B). The tissue distribution results showed that *gcFGF8a* was widely distributed and highly expressed in the mucosal immune organs, especially the skin (Figure 1C). Accordingly, after bacterial infection, *gcFGF8a* expression increased in the mucosal immune organs but not the systemic immune organs (Figure 1D,E). By prokaryotic expression and purification, we obtained gcFGF8a protein (Figure 1F) and found that injection of gcFGF8a protein can reduce the mortality and skin bacterial load of grass carp caused by *A. hydrophila* (Figure 1G,H). These results indicated that gcFGF8a participates in the antibacterial immunity of grass carp.

### 2.2 gcFGF8a‐Induced Membrane‐Dependent Bacterial Killing

**Figure 2:**
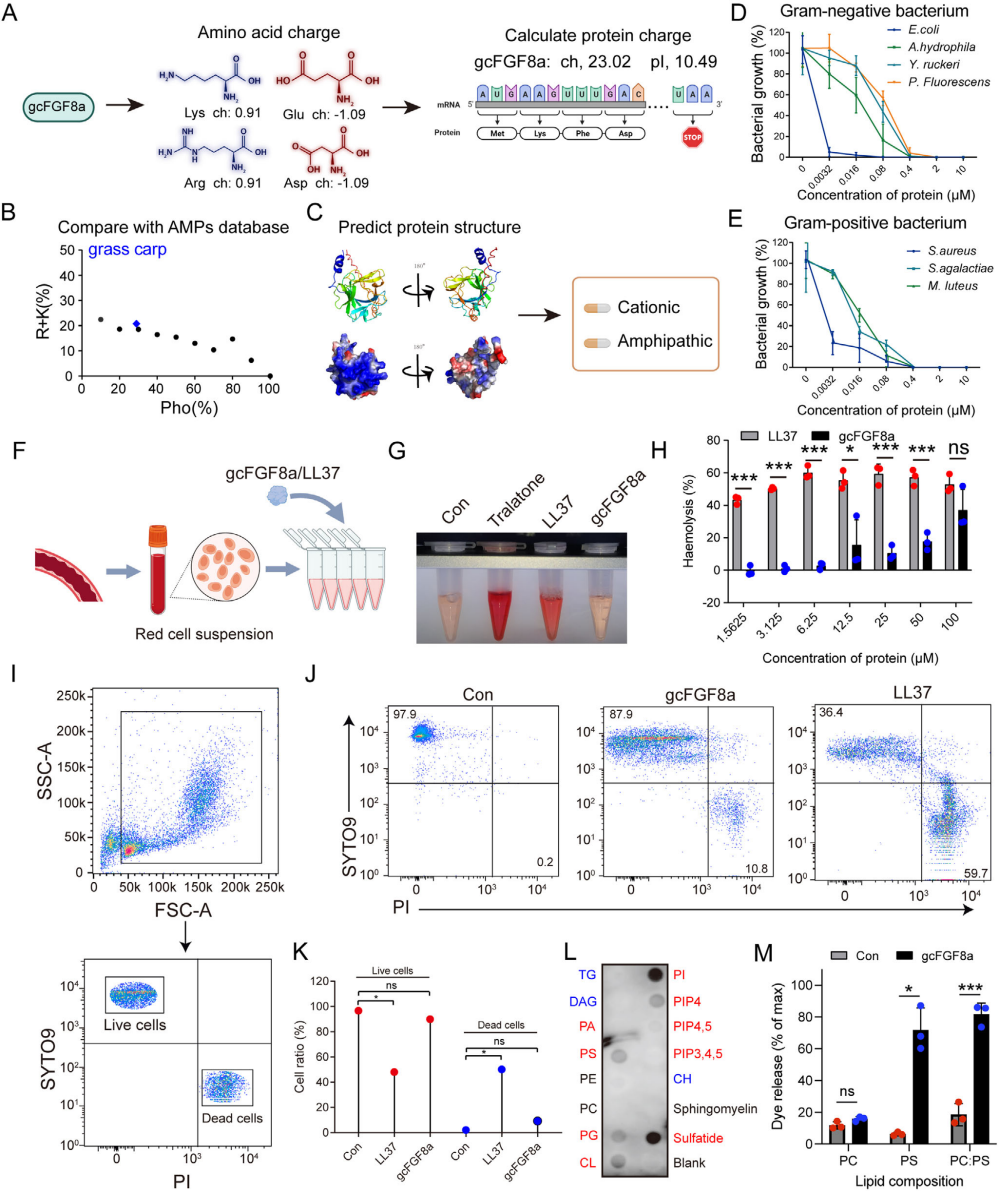
gcFGF8a has broad‐spectrum antibacterial activity and low host toxicity. A) The isoelectric point and charge distribution are determined using DNASTAR software. B) A (R+K)%‐Pho% dot plot compares gcFGF8a to AMPs from the AMP database. The 2339 AMPs in the AMP database were separated into 10 bins based on the hydrophobic contents (0–10, 11–20, 21–30, 31–40, 41–50, 51–60, 61–70, 71–80, 81–90, and 91–100%), which are represented as 10, 20, 30, 40, 50, 60, 70, 80, 90, and 100, respectively, on the x‐axis of the plot. Τhe AMPs from the database are depicted in black, with gcFGF8a highlighted in blue. C) Representation of the tertiary structure and surface electrostatic potential of gcFGF8a. The positively charged (blue), negatively charged (red), and hydrophobic (white) residues are highlighted. D,E) Bactericidal activity of gcFGF8a against Gram‐negative and positive bacteria. The CFU assay was conducted, and the treated bacteria were spread onto TSA plates, incubated, and then counted, *n* = 3. F) Schematic representation of hemolytic toxicity assay. G) Image demonstrating the hemolytic effect of 4 µM gcFGF8a, using LL37 as a positive control. H) Hemolytic toxicity of different concentrations of gcFGF8a and LL37. The percentage of hemolysis was calculated through measuring the OD_405_ of the supernatant after 2 h incubation, *n* = 3. I) Schematic diagram of the cytotoxicity assay by flow cytometry. Grass carp HKLs were co‐stained with PI and SYTO9, then live and dead cells were defined as SYTO9^+^ PI^–^ and SYTO9^–^ PI^+^, respectively. (J) Detection of cytotoxicity of gcFGF8a. Image demonstrated the cytotoxicity of 4 µM gcFGF8a, with LL37 as a positive control. K) Statistical analysis of gcFGF8a cytotoxicity based on data from three replications of (J), *n* = 3. L) Binding activity of gcFGF8a to different lipids. Membrane‐containing lipids were incubated with 1 µg mL^−1^ gcFGF8a and assayed with His mAbs. Red represents negatively charged lipids, blue represents positively charged lipids, and black represents neutral lipids. M) Carboxyfluorescein (CF)–loaded liposomes with the indicated lipid compositions were treated with 1 µg mL^−1^ gcFGF8a. Total dye release was determined by adding 1% OG, *n* = 3. *p ≤ 0.05, **p ≤ 0.01, ***p ≤ 0.001.

Biochemical analysis revealed that gcFGF8a is a basic protein (pI 10.49) with a high positive charge (+23.02) (**Figure** **2**A). The distribution of hydrophobic amino acids and basic amino acids of gcFGF8a is consistent with known AMPs in the AMPs database (Figure 2B). The tertiary structure prediction model reveals that gcFGF8a predominantly consists of β sheets, α helices, and random coils. The positively charged amino acids cluster on one side of the molecule's surface, while the other side is composed of negatively charged and hydrophobic amino acid, forming a hydrophobic surface (Figure 2C). Thus, gcFGF8a is a cationic and amphipathic molecule like classical AMPs.^[^ ^25^ ^]^ Subsequently, we found that gcFGF8a exhibits broad‐spectrum and potent antibacterial activity against both Gram‐negative and Gram‐positive bacteria (Figure 2D,E). Unlike LL37, which has high cytotoxicity, gcFGF8a exhibits lower hemolysis (Figure 2F–H) and cytotoxic effects (Figure 2I–K). This may be mainly attributed to gcFGF8a's selective targeting and disruption of liposomes containing acidic lipids, but not those containing zwitterionic lipids (Figure 2L,M). Compared to eukaryotic membranes composed of zwitterionic lipids (phosphatidylcholine), bacterial membranes contain a variety of acidic phospholipids (phosphatidylglycerol, phosphatidylserine, and cardiolipin).^[^ ^27^ ^]^

**Figure 3:**
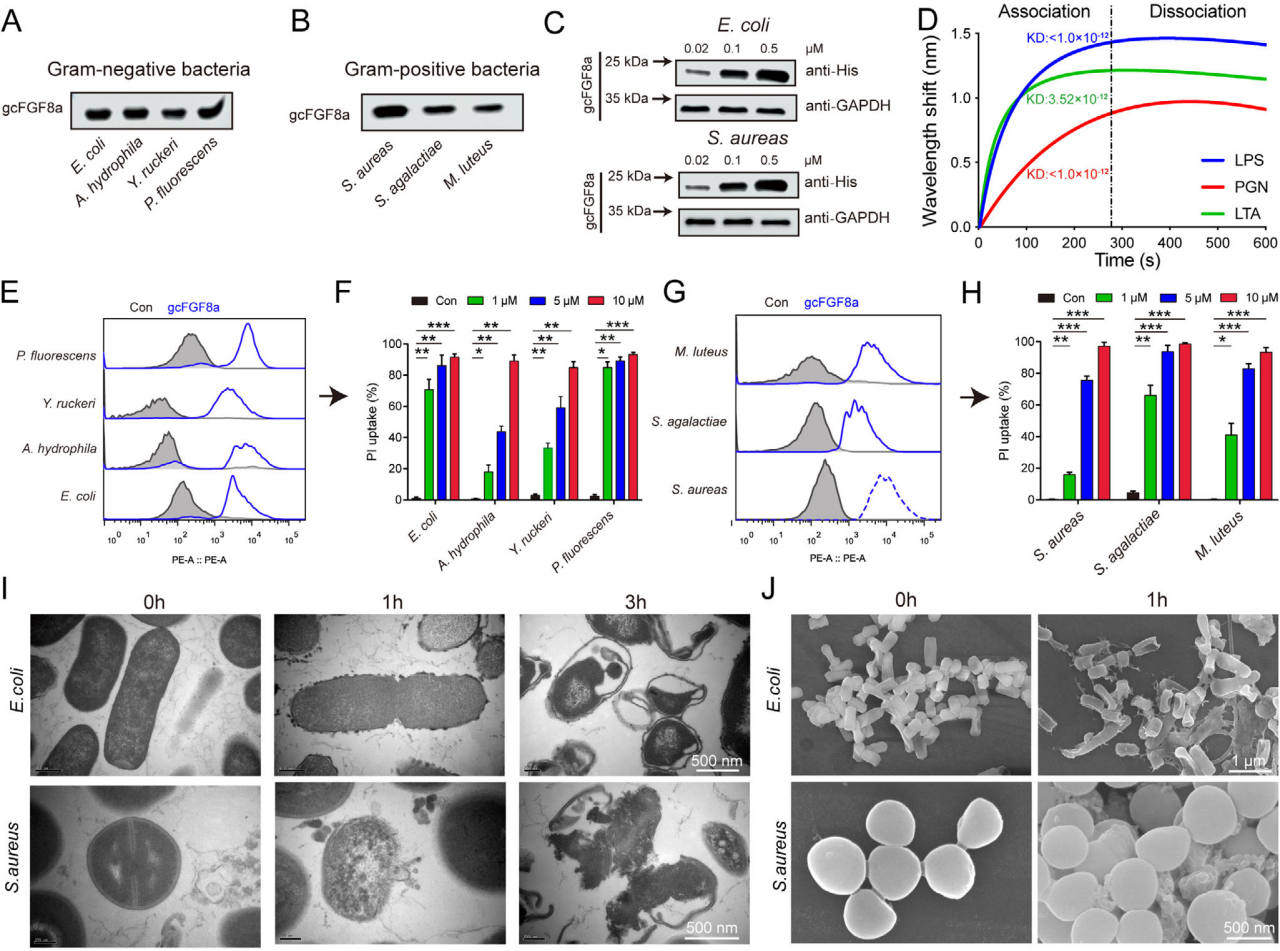
gcFGF8a kills Gram‐negative and positive bacteria in a membrane‐dependent manner. A,B) Binding activity of gcFGF8a to Gram‐negative and positive bacteria. gcFGF8a (0.1 µM) was incubated with Gram‐negative (A) and Gram‐positive (B) bacteria at 37 °C for 1 h, and the presence of gcFGF8a bound to the bacteria was detected using Western blot analysis. C) Concentration‐dependent binding activity of gcFGF8a to *E. coli* and *S. aureus*. Similarly, the binding activity was detected using Western blot analysis. D) Binding activity of gcFGF8a to PAMPs. Association and dissociation curves of gcFGF8a to PAMPs were recorded by bio‐layer interferometry. E–H) Quantitative analysis of bacterial membrane permeability. The PI uptake of Gram‐negative (E) and Gram‐positive (G) bacteria significantly increased after treated with 10 µM gcFGF8a for 1 h. Data analysis of PI uptake assay for membrane integrity of Gram‐negative (F) and Gram‐positive (H) bacteria treated with gcFGF8a at different concentrations, *n* = 3. I,J) TEM and SEM imaging of *E. coli* and *S. aureus*. Following treatment with gcFGF8a, *E. coli* and *S. aureus* were fixed for ultrastructural examination. *p ≤ 0.05, **p ≤ 0.01, ***p ≤ 0.001.

To elucidate the antibacterial mechanism of gcFGF8a, we investigated the binding ability of recombinant gcFGF8a to bacteria and PAMPs. As shown in **Figure** **3**A,B, recombinant gcFGF8a could bind to various bacteria, including four Gram‐negative bacteria (*E. coli*, *A. hydrophila*, *Y. ruckeri*, and *P. fluorescens*) and three Gram‐positive bacteria (*S. aureus*, *S. agalactiae*, and *M. luteus*). Using *E. coli* and *S. aureus* as representatives, we discovered that the binding activity of recombinant gcFGF8a to bacteria was positively correlated with its concentration (Figure 3C). To explore the underlying mechanism behind the broad‐spectrum binding activity of gcFGF8a to bacteria, we examined the binding activity of gcFGF8a to various PAMPs (LPS, PGN, and LTA). As shown in Figure 3D and Figure S3 (Supporting Information), gcFGF8a exhibited picomolar binding affinity and concentration‐dependent binding activity for LPS, PGN, and LTA. Moreover, gcFGF8a exerted killing effect on both Gram‐negative and Gram‐positive bacteria in a membrane‐dependent manner. This was supported by the PI uptake assay, which showed that gcFGF8a induced permeability changes in bacterial membrane of both Gram‐negative and Gram‐positive bacteria (Figure 3E–H; Figure S4, Supporting Information). TEM and SEM also revealed membrane damage and cell contents leakage in both Gram‐negative and Gram‐positive bacteria after the treatment of gcFGF8a (Figure 3I,J).

### 2.3 Antibacterial and Skin Wound Repair Functions of gcFGF8a

**Figure 4:**
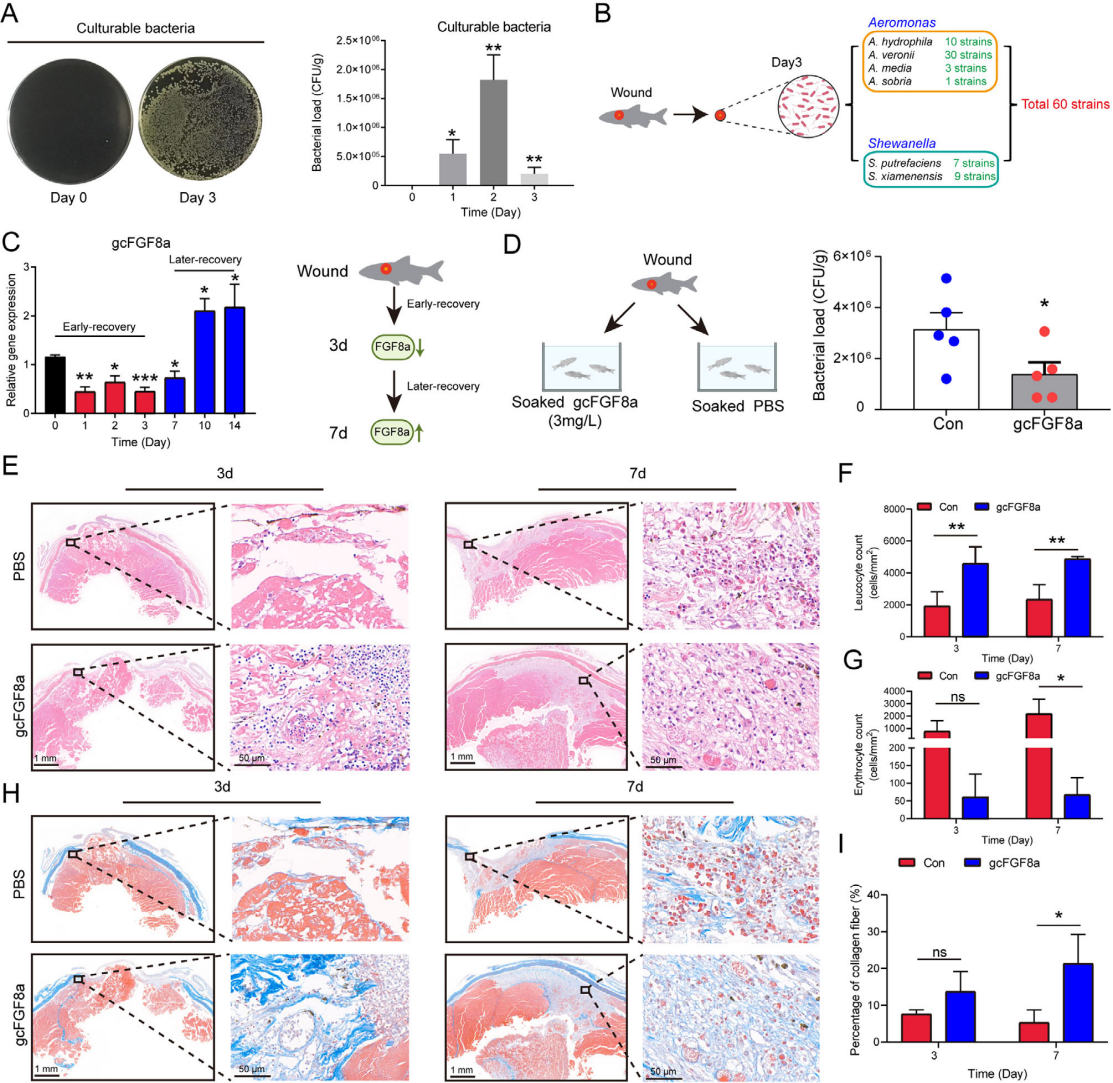
Antibacterial and pro‐healing effects of gcFGF8a in wound areas. A,B) Bacterial load at the wound site after trauma was determined. The bacterial load in the wound tissue was determined by TSA plating, *n* = 5. B) The identification of bacterial species in the wound was conducted. A total of 60 strains were randomly isolated from the TSA agar plate and then identified by 16S rDNA sequencing. C) The dynamic expression of *gcFGF8a* after trauma. The expression of *gcFGF8a* after trauma was determined by RT‐qPCR, with the *18S rRNA* gene as the internal control, *n* = 5 (left). Diagram illustrating the expression pattern of *gcFGF8a* after trauma (right). D) The changes of the level of *Aeromonas* in wounds following gcFGF8a treatment. Schematic representation of gcFGF8a treatment of trauma (left). The *Aeromonas* in wound was counted using *Aeromonas* spp. selective plates, *n* = 5 (right). E) HE staining results of wound tissue in control and gcFGF8a treatment group after trauma. The wound tissues were collected at 3 and 7 days after trauma to evaluate tissue damage. F,G) Quantification of leukocyte and erythrocyte content in the wounds in control and gcFGF8a‐treated groups using Image Pro Plus software. Statistics were performed on 5 randomly selected areas. H) Masson's trichrome staining of wound tissue in different groups. The wound tissues were collected at 3 and 7 days after trauma to examine collagen fiber accumulation. I) Quantification of collagen fiber densities in wound tissue in different groups. Statistics were performed on 5 randomly selected areas. *p ≤ 0.05, **p ≤ 0.01, ***p ≤ 0.001.

In general, skin damage in fish is negatively correlated with their disease resistance.^[^ ^28^ ^]^ Consistent with this, we found that after skin wounds, the bacterial load at the wound site significantly increased in grass carp (**Figure** **4**A), especially the pathogenic bacteria *Aeromonas* (Figure 4B). Subsequently, as shown in Figure S5A,B (Supporting Information), HE staining revealed severe tissue damage and bleeding after skin wounds, consistent with the sepsis symptoms induced by *Aeromonas*.^[^ ^29^, ^30^ ^]^ Masson trichrome staining demonstrated the collagen fibers deposition in the wound sites 7 days after skin trauma, indicating the initiation of wound repair (Figure S5C,D, Supporting Information). Correspondingly, we observed scarce gcFGF8a expression in the early stages of trauma, but gradually recovered and even exceeded the initial levels during the later stages of wound healing (Figure 4C). These findings suggesting that gcFGF8a may play roles in the process of skin wound healing.

**Figure 5:**
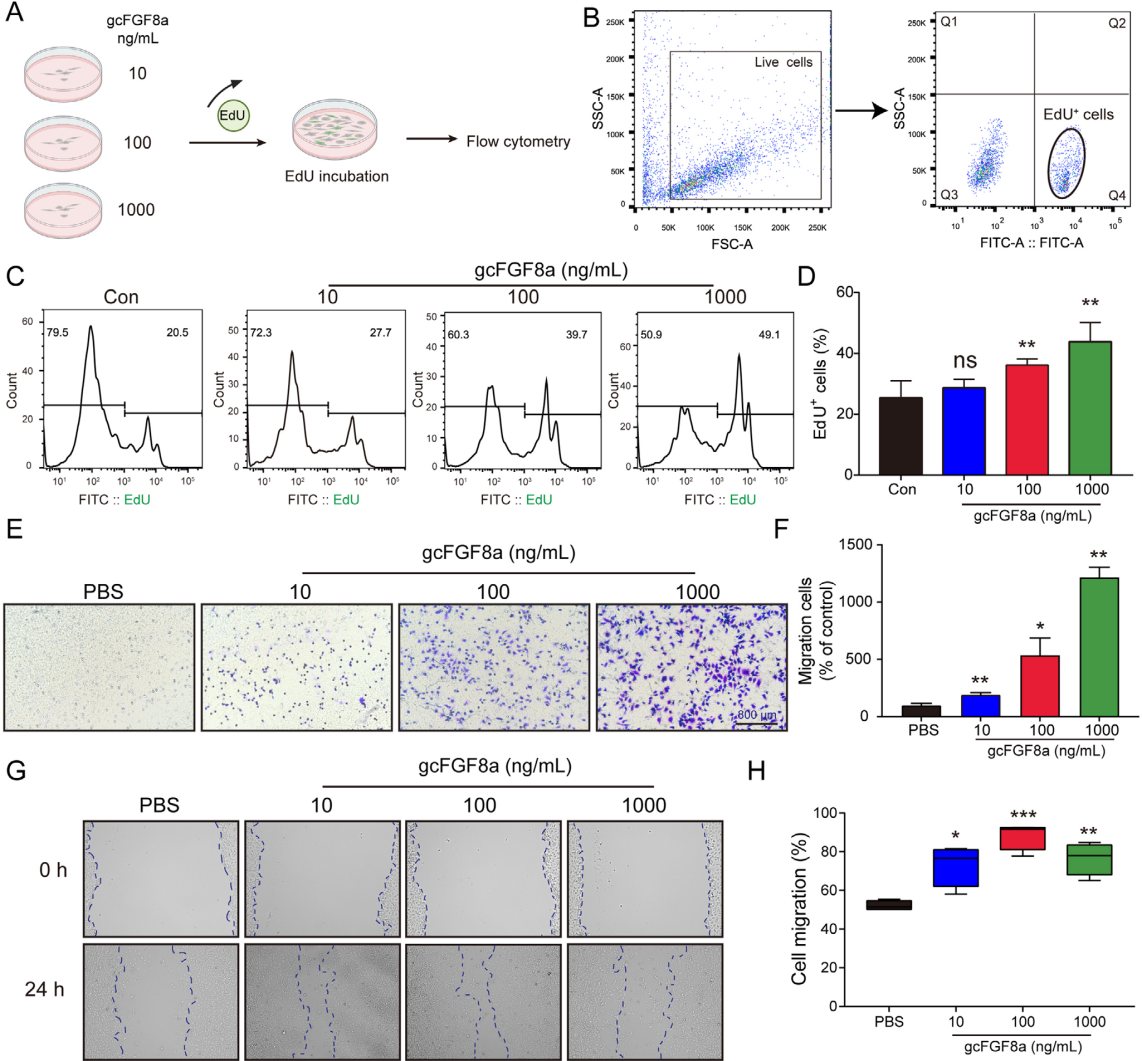
Regulation of EPC proliferation and migration by gcFGF8a. A) Schematic diagram of the cell proliferation assay. EPCs were co‐incubated with different concentrations of gcFGF8a and 1 µM EdU. B) Schematic diagram of cell proliferation detected by flow cytometry. EdU^+^ cells were considered proliferative. C) gcFGF8a promotes epithelial cell proliferation in a concentration‐dependent manner. Flow cytometry peak plot illustrating the pro‐proliferative effect of different concentrations of gcFGF8a on EPCs. D) The data from (C) are presented as means ± SD, with 5 repetitions. E) Effects of different concentrations of gcFGF8a on EPC cell migration. For the negative control, the lower chambers were filled with PBS. F) Quantitative statistics of cell migration in different groups, *n* = 3. G) The scratch assay was employed to evaluate cell migration and wound recovery. Blue dotted lines indicate the scratched edges. H) Quantitative statistics of cell migration in different groups. The scratch area was calculated using Image Pro Plus software, and quantified as a percentage relative to the start area at 0 h, *n* = 4. *p ≤ 0.05, **p ≤ 0.01, ***p ≤ 0.001.

Since the load of *Aeromonas* in wounds increased after skin trauma, we evaluated the relationship between *Aeromonas* infection and wound repair. Our investigations showed that *A. hydrophila* infection could exacerbate tissue damage and delay wound healing (Figure S6A,B, Supporting Information). To evaluate the potential of gcFGF8a to improve wound healing, gcFGF8a protein (3 mg L^−1^) was administered to grass carp with skin trauma via immersion (Figure 4D). Consistent with the in vitro antibacterial activity, gcFGF8a could function as an AMP to maintain flora homeostasis, because gcFGF8a immersion reduced the bacterial loads of *Aeromonas* at the wound site (Figure 4D). HE staining revealed that gcFGF8a immersion facilitated leukocyte enrichment (Figure 4E,F), and decreased bleeding (Figure 4E,G). Masson's trichrome staining indicated that compared to the control group, the gcFGF8a‐treated group exhibited enhanced collagen deposition and improved organization of fibrous tissue (Figure 4H,I). These findings collectively suggest that besides its antibacterial function, gcFGF8a has the ability to promote wound repair. To elucidate the cellular mechanisms underlying the pro‐repair effects of gcFGF8a, we evaluated its activity on epithelial cells (EPCs) in vitro. Our results revealed that gcFGF8a can promote the proliferation and migration of EPCs in the concentration‐dependent manna (**Figure** **5**A–F), thereby accelerating wound healing in scratch assays (Figure 5G,H). These results directly demonstrated the ability of gcFGF8a to promote cellular repair processes.

### 2.4 Bacterial Infection Triggers Wnt‐Dependent gcFGF8a Expression to Activate FGFR4‐ERK/AKT‐mTORC1‐HIF1α Signaling Axis in Wound Repair

**Figure 6:**
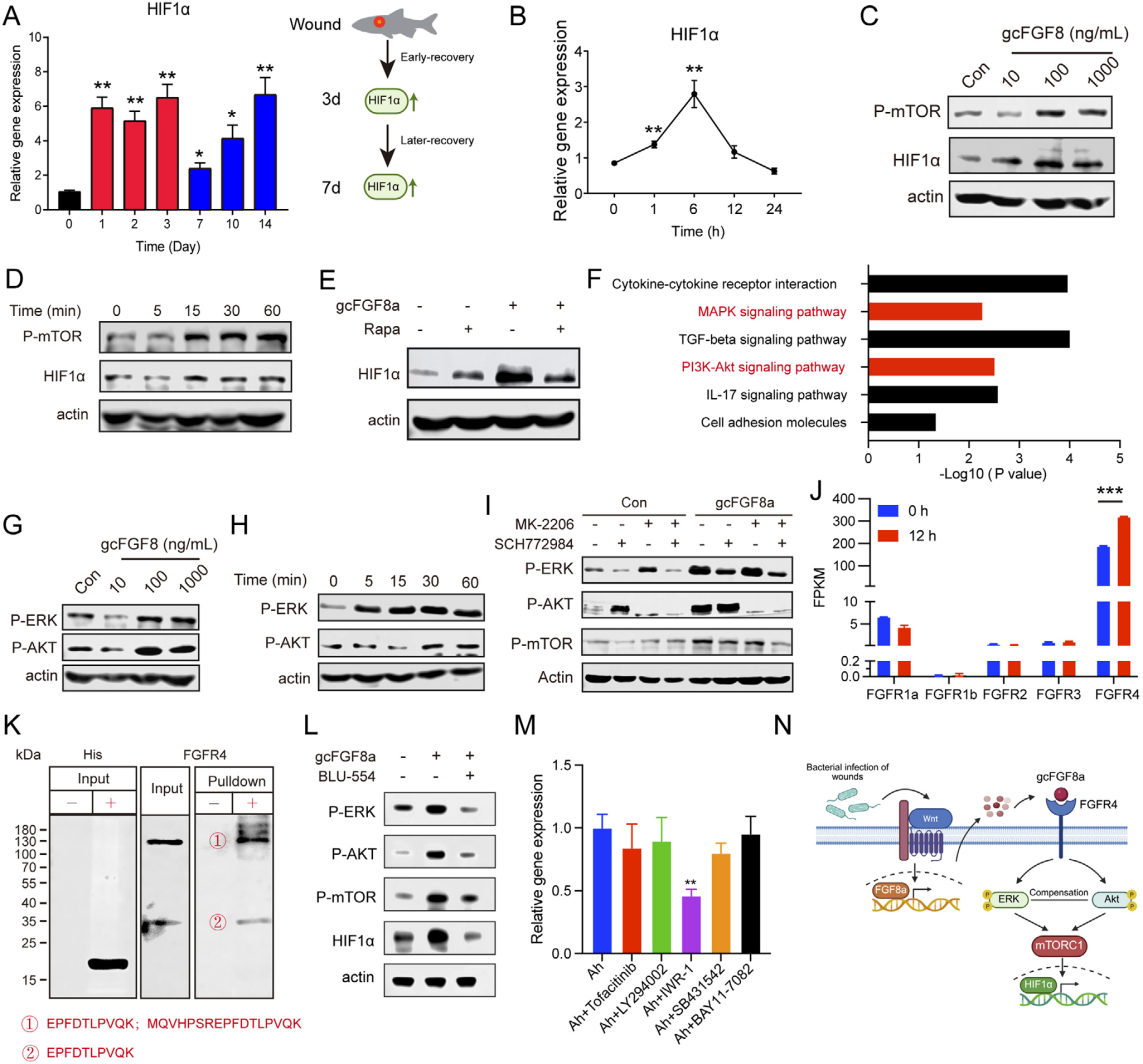
ERK/AKT signaling downstream of gcFGF8a controls mTOR and HIF1a. A) The expression changes of *HIF1α* after trauma were detected by RT‐qPCR. The fold changes were normalized to the *18S rRNA* gene, *n* = 5. B) Effect of gcFGF8a on *HIF1α* expression in EPCs. *HIF1α* expression was assessed by RT‐qPCR at various time points after stimulation with 100 ng mL^−1^ gcFGF8a, *n* = 5. C) gcFGF8a promotes concentration‐dependent *HIF1α* expression and mTOR phosphorylation. Western blot was carried out to detect the *HIF1α* expression and mTOR phosphorylation levels after 1 h treatment. D) Impact of different durations of gcFGF8a treatment on *HIF1α* expression and mTOR phosphorylation. Western blot was used to detect the effects. E) Rapamycin attenuates gcFGF8a‐mediated *HIF1a* expression. Rapamycin (100 nM) was used to inhibit mTOR phosphorylation. F) Enriched KEGG pathways after gcFGF8a treatment. G) Effects of different concentrations of gcFGF8a on mTOR proximal signaling ERK/AKT. H) Impact of varying durations of gcFGF8a treatment on ERK/AKT phosphorylation. I) Inhibition of ERK and/or AKT impedes gcFGF8a‐induced mTOR phosphorylation. SCH772984 (1 µM) and MK‐2206 (5 µM) were used to inhibit ERK and AKT, respectively. J) The expression levels of FGFRs in EPCs. FPKM represents the gene abundance in the sequenced data of the transcriptome libraries. K) His‐ pull‐down assay showing the interaction of His‐gcFGF8a to FGFR4 in EPCs cell lysate. “‐” represents empty Ni‐NTA agarose beads, and “+” represents agarose beads bound with gcFGF8a. L) Inhibition of FGFR4 impedes gcFGF8a‐induced mTOR phosphorylation. BLU‐554 (5 nM) was used to inhibit FGFR4. M) Inhibition of Wnt suppresses gcFGF8a expression after bacterial infection. BAY11‐7082 (1 µM), Tofacitinib (1 µM), LY294002 (20 µM), SB431542 (10 µM), and IWR‐1 (5 µM) were used to inhibit NF‐κB, JAK, PI3K, TGF‐β, and Wnt respectively. N) Schematic representation illustrating the mechanism of gcFGF8a promoting *HIF1α* expression. *p ≤ 0.05, **p ≤ 0.01, ***p ≤ 0.001.

Research has established that acute skin injuries typically necessitate the adaptation to a locally hypoxic microenvironment mediated by HIF1α.^[^ ^31^, ^32^ ^]^ In this study, we found that teleost HIF1α also participates in acute skin injuries because the expression of grass carp *HIF1α* escalated within 3 days after skin trauma (**Figure** **6**A). Notably, at day 7 after skin trauma, both the expression of *HIF1α* and *gcFGF8a* escalated synchronously (Figures 4C and 6A), promoting the speculation that gcFGF8a may be associated with hypoxia response during the wound healing process. Moreover, gcFGF8a stimulation increased HIFα expression at mRNA and protein levels (Figure 6B–D). This further suggests that the gcFGF8a‐HIIFα pathway may be involved in wound healing. To elucidate the specific mechanisms, we evaluated whether mTOR, a regulator of HIF1α,^[^ ^33^, ^34^ ^]^ could modulate the gcFGF8a‐HIF1α pathway. Given the high similarity between grass carp mTOR, and their human counterparts (Figure S7, Supporting Information), we used human pmTOR‐specific antibodies to assess phosphorylation changes of the proteins after FGF8a stimulation. Our findings revealed that gcFGF8a could enhance mTOR phosphorylation 15 min after stimulation, with an optimal concentration of 100 ng mL^−1^ (Figure 6C,D). Furthermore, inhibition of mTOR by rapamycin could attenuate gcFGF8a‐induced upregulation of HIF1α (Figure 6E), suggesting that gcFGF8a can promote HIF1α expression via mTOR phosphorylation.

To elucidate the upstream signaling mechanisms, we performed RNA‐seq analysis on EPCs stimulated with gcFGF8a. KEGG pathway enrichment analysis revealed significant upregulation of genes in the MAPK and PI3K‐AKT pathways (Figure 6F), both of which are known to regulate mTOR.^[^ ^33^, ^35^, ^36^ ^]^ Due to the high sequence similarity between grass carp and human ERK/AKT (Figure S7, Supporting Information), we used human‐specific pERK/pAKT antibodies to assess phosphorylation changes in grass carp ERK/AKT after FGF8a stimulation. We observed that gcFGF8a stimulation could promote the phosphorylation of ERK and AKT, upstream of mTOR, within 1 h (Figure 6G,H). Notably, inhibition of ERK lead to compensatory increases in p‐AKT, and vice versa (Figure 6I). Only simultaneous inhibition of ERK and AKT could effectively inhibit mTOR phosphorylation downstream of gcFGF8a (Figure 6I), indicating that gcFGF8a activates mTOR through both ERK and AKT.

Subsequently, we identified FGFR4 as the obligatory receptor for gcFGF8a‐induced ERK and AKT phosphorylation in EPCs. RNA‐seq analysis revealed FGFR4 as the most abundant FGFR family member in EPC cells, with expression increased upon gcFGF8a stimulation (Figure 6J). Pull‐down assay confirmed direct binding between gcFGF8a and FGFR4, with mass spectrometry identifying FGFR4 as the exclusive FGFR family member interacting with gcFGF8a (Figure 6K). Furthermore, FGFR4 inhibitor suppressed gcFGF8a‐induced ERK/AKT/mTOR phosphorylation and HIF1α expression (Figure 6L).

Bacterial infection emerged as a critical driver of gcFGF8a expression, with significant upregulation observed in infected wounds and post‐infection tissues, highlighting a strong correlation between bacterial infection and gcFGF8a induction (Figures 1B,D and 4C). To elucidate the potential mechanisms of gcFGF8a induction, we screened inhibitors targeting pathways classically activated by bacterial infection, including Tofacitinib (JAK), LY294002 (PI3K), IWR‐1 (Wnt), SB431542 (TGF‐β), and BAY11‐7082 (NF‐κB). Notably, inhibition of Wnt signaling using IWR‐1, but not other pathway inhibitors, significantly attenuated gcFGF8a upregulation after *A. hydrophila* infection, implicating Wnt is the primary mediator of infection‐induced gcFGF8a expression (Figure 6M).

Collectively, these data define a multi‐tiered signaling cascade where bacterial infection activates Wnt to induce gcFGF8a, which binds to FGFR4 to drive ERK/AKT‐mTORC1‐HIF1α signaling, thereby creating a pro‐repair environment (Figure 6N).

### 2.5 gcFGF8a‐HIF1α Axis Promotes Wound Healing Through Glycolysis

**Figure 7:**
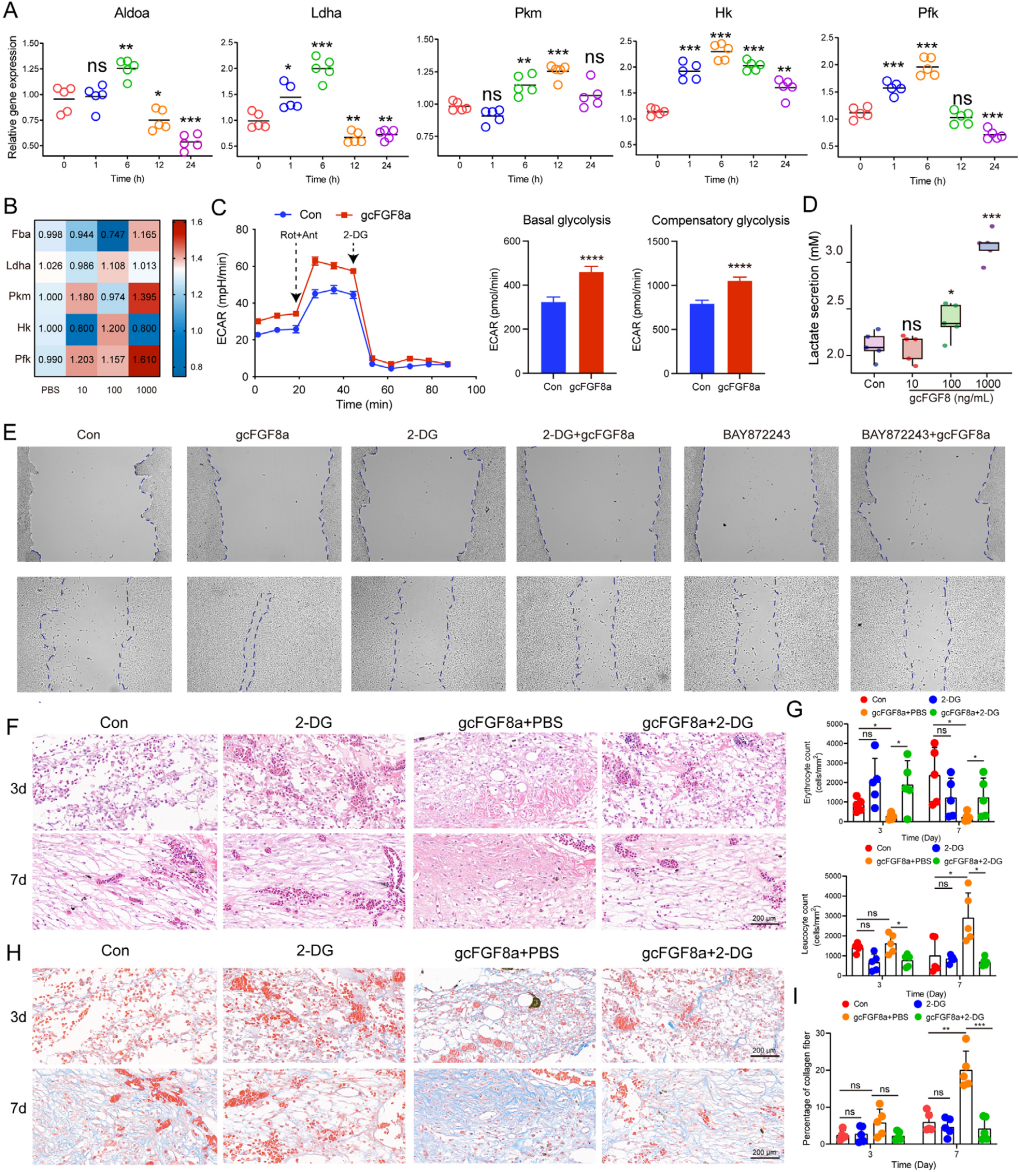
gcFGF8a‐HIF1a‐mediated glucose metabolism promotes wound repair. A) gcFGF8a enhances the expression of glycolysis‐related genes in EPCs. RT‐qPCR analysis of *Aldoa*, *Ldha*, *Pkm*, *Hk*, and *Pfk* expression at different time points after stimulating cells with 100 ng mL^−1^ gcFGF8a, *n* = 5. B) Augmented glycolytic enzyme activity in EPCs following gcFGF8a stimulation. Enzyme activities were assayed using appropriate kits after 24 h stimulation, *n* = 5. C) The rate of cellular glycolysis. The basal glycolysis and the compensatory glycolysis of mitochondria were measured. *n* = 6. D) gcFGF8a promotes the secretion of the glucose metabolite lactate. Extracellular lactic acid secretion was detected using the lactic acid assay kit, *n* = 5. E) Inhibition of HIF1α or glycolysis suppresses the promotion of EPCs migration by gcFGF8a. BAY872243 and 2‐DG were used to inhibit HIF1α and glycolysis, respectively. F) Glycolysis inhibition abrogates the promotion of tissue repair by gcFGF8a. Using HE staining to examine the tissue damage. G) Statistical analysis of erythrocyte and leukocyte content in wounds. The data was performed on 5 randomly selected areas. H) Glycolysis inhibition attenuates the promotion of tissue collagen fiber formation by gcFGF8a. Using Masson's trichrome staining to examine the collagen fiber accumulation. I) Statistical analysis of collagen fiber densities in wounds. The data was performed on 5 randomly selected areas. *p ≤ 0.05, **p ≤ 0.01, ***p ≤ 0.001.

Migration is a pivotal aspect of re‐epithelialization during wound healing, necessitating cellular energy expenditure.^[^ ^1^, ^19^ ^]^ Glycolysis is one of the major pathways for cellular energy production, with HIF1α serving as a key regulator.^[^ ^37^ ^]^ Therefore, the upregulation of HIF1α expression by gcFGF8a suggests its potential involvement in promoting wound repair through glycolysis. As shown in Figure S8A–F (Supporting Information), the expression levels of glycolysis‐related genes, *Aldoa* (*Aldoase A*), *Ldha* (*Lactate dehydrogenase A*), *Pkm* (*Pyruvate kinase M*), and *HK* (*hexokinase*), except *Pfk* (*Phosphofructokinase*), decreased during the initial three days after skin trauma, highlighting the intimate connection between glycolysis and wound repair. In vitro, gcFGF8a stimulation enhanced the expression of glycolysis enzyme genes in EPCs (**Figure** **7**A). In addition, compared with the control group, EPCs treated with gcFGF8a exhibited heightened glycolytic enzyme activity, enhanced basal and compensatory glycolysis, and secreted elevated levels of lactate (the end product of glycolysis) (Figure 7B–D).

To further confirm the potential of the gcFGF8a‐HIF1α axis in promoting epithelial cell migration through glucose metabolism, we investigated the effects of a glycolysis inhibitor (2‐DG) and a HIF1α inhibitor (BAY872243) on gcFGF8a‐induced cell migration in vitro. The epithelial scratch assay demonstrated that both the glycolysis inhibitor and the HIF1α inhibitor could attenuate gcFGF8a‐induced migration in EPCs (Figure 7E). Similarly, in vivo, the use of 2‐DG could counteract the pro‐healing effect of gcFGF8a. HE staining showed that compared with gcFGF8a group, the 2‐DG group exhibited reduced leukocyte infiltration and increased bleeding at the wound site (Figure 7F,G). Additionally, Masson's trichrome staining showed decreased collagen content and irregular fibrous tissue structure in the 2‐DG group (Figure 7H,I). These findings collectively suggest that gcFGF8a can accelerate wound healing by enhancing the glycolytic metabolic pathway.

### 2.6 mFGF8b, the Homolog of gcFGF8a, also Maintains Microbial Balance and Enhances Glycolysis to Accelerate Wound Healing

**Figure 8:**
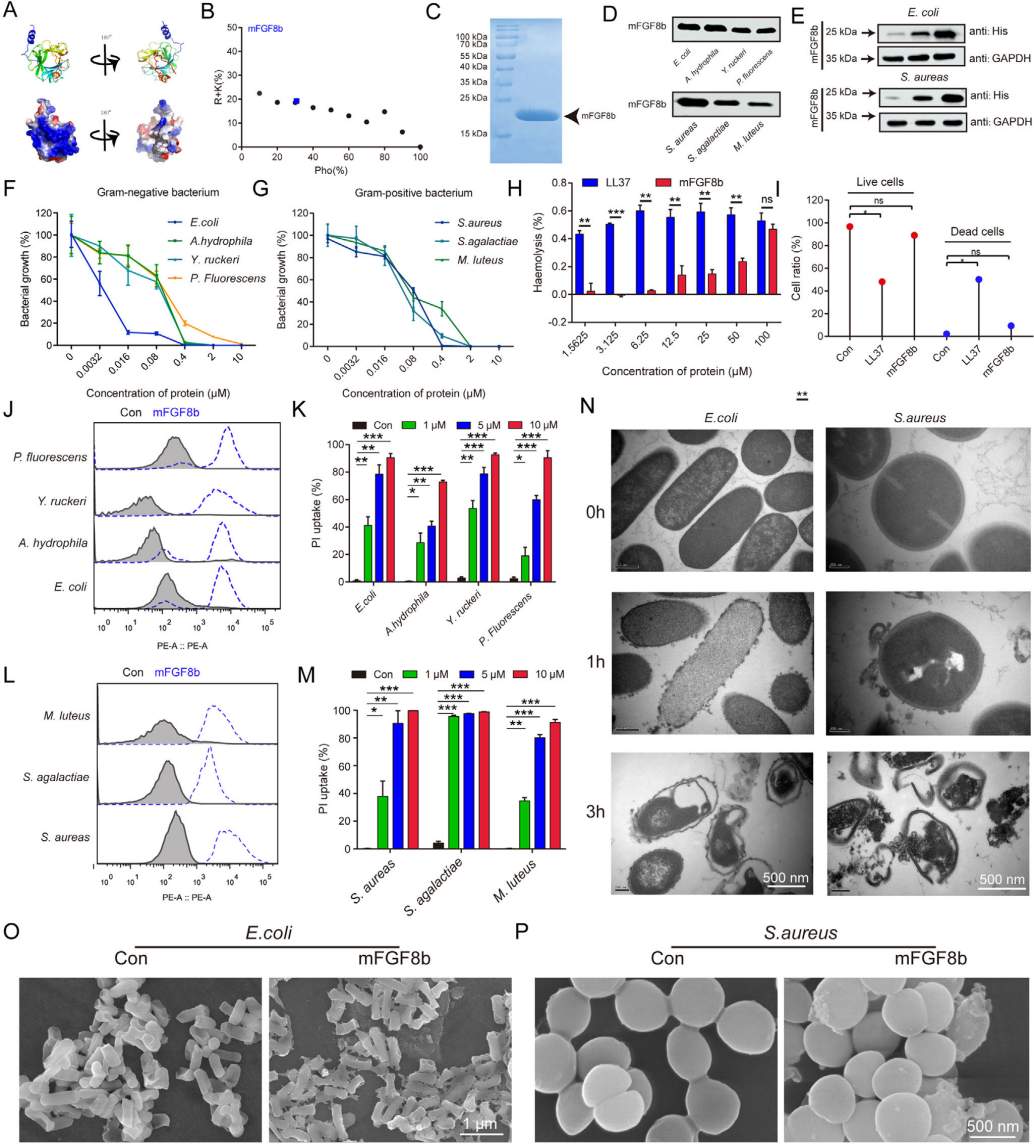
mFGF8b also exhibits broad‐spectrum bactericidal activity. A) The tertiary structure of mFGF8b (upper panel) and the electrostatic sites displayed on the molecular surface (lower panel). B) A (R+K)%‐Pho% dot plot compares mFGF8b to AMPs from the AMP database. C) SDS‐PAGE analysis of recombinant mFGF8b purified from *E. coli*. D) Binding activity of mFGF8b with Gram‐negative and positive bacteria. E) Concentration‐dependent binding activity of mFGF8b to *E. coli* and *S. aureus*. F‐G) Bactericidal activity of mFGF8b assessed by CFU counting method, *n* = 3. (H) mFGF8b has low hemolytic toxicity compared to LL37, *n* = 3. I) Low cytotoxicity of mFGF8b compared to LL37 assessed by PI and SYTO9 co‐staining, *n* = 3. J) Membrane permeability of *E. coli* after mFGF8b treatment. Analysis by flow cytometry using PI as an indicator. K) Effect of different concentrations of mFGF8b on *E. coli* membrane permeability, *n* = 3. L) Membrane permeability of *S. aureus* after mFGF8b treatment. M) Effect of different concentrations of mFGF8b on *S. aureus* membrane permeability, *n* = 3. N–P) TEM and SEM imaging of *E. coli* and *S. aureus*. After treating *E. coli* (N and O) and *S. aureus* (N,P) with mFGF8b for different times, they were fixed for ultrastructural observation.

Studies have shown that mFGF8 has 8 variants (a‐h),^[^ ^26^ ^]^ among which isoforms mFGF8a, b, c, d share the same gene structure with gcFGF8a, containing 5 exons and 4 introns (Figure S9A, Supporting Information). Multiple sequence alignment analysis revealed a higher similarity between mFGF8b and gcFGF8a (Figure S9B, Supporting Information). Like gcFGF8a, mFGF8b is also a basic protein (pI 10.49) with a high positive charge (ch, +19.86). The distribution of hydrophobic amino acids and basic amino acids (K and R) of mFGF8b is consistent with known AMPs in the AMP database (**Figure** **8**A,B).^[^ ^25^ ^]^ Similar to gcFGF8a, purified mFGF8b protein could also directly target various Gram‐negative and Gram‐positive bacteria (Figure 8C–E), exhibiting broad‐spectrum antibacterial activity and good biocompatibility (Figure 8F–I). PI uptake assay as well as TEM and SEM observations revealed that mFGF8b could kill Gram‐positive and Gram‐negative bacteria through a membrane‐dependent mechanism (Figure 8J–P).

**Figure 9:**
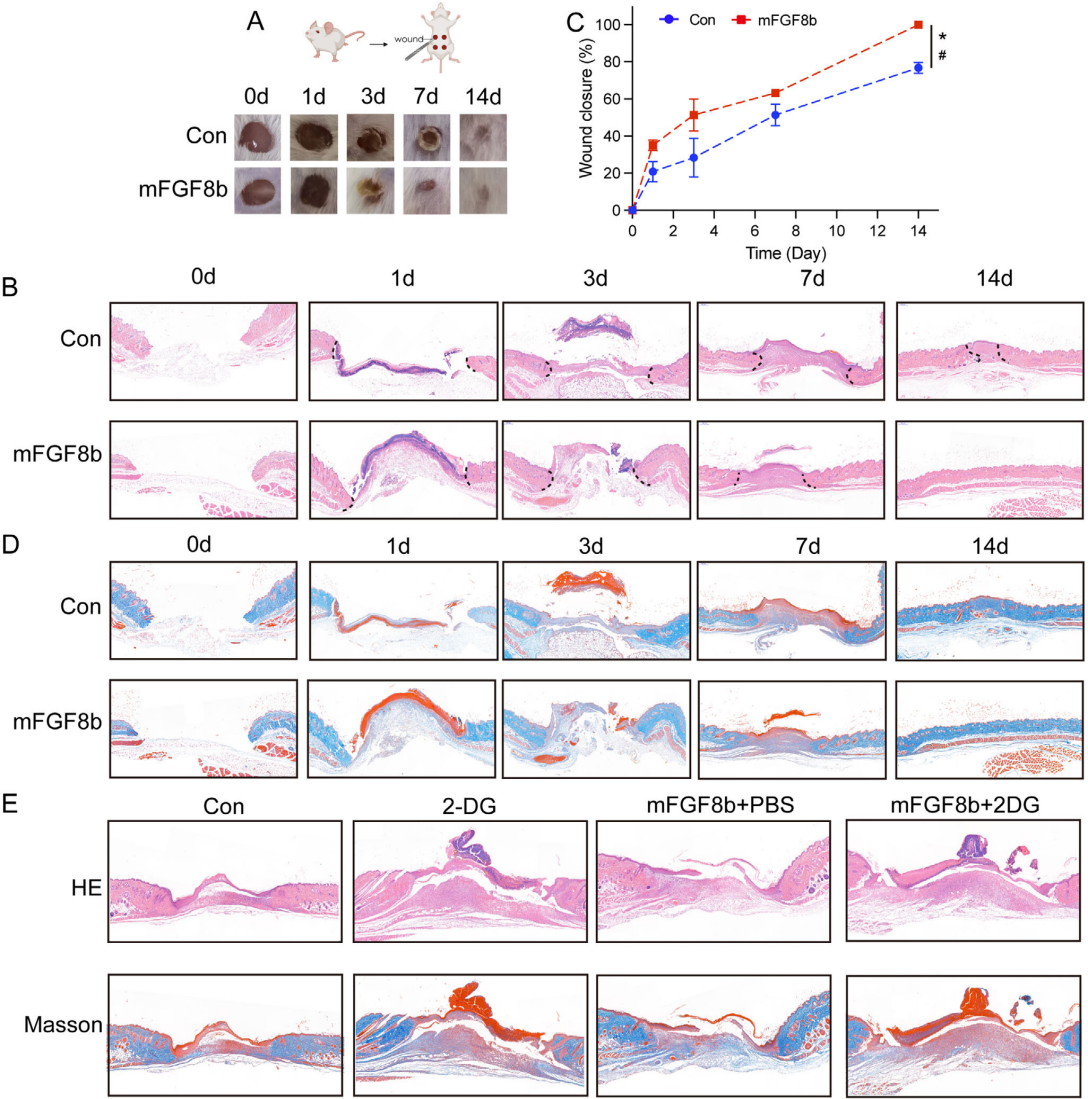
mFGF8b promotes wound repair through glycolysis in mice. A) Representative images of skin wounds in each group at days 0, 1, 3, 7, and 14 after trauma. B) HE staining results in wound tissue after trauma in different groups. After coating mFGF8b externally, HE staining was used to examine the tissue damage. C) Wound repair rates in different groups. Repair rates were determined using Image Pro Plus software by measuring wound gap distances at three randomized positions per histological section across three independent sections per group. The data was expressed as a percentage of the initial wound area at 0 h. D) Masson's trichrome staining of wound samples. After coating mFGF8b externally, Masson's trichrome staining was used to examine collagen fiber accumulation. E) Inhibition of glycolysis inhibits tissue repair and collagen fiber accumulation. mFGF8b, glycolysis inhibitor (2‐DG) or an equal volume of PBS was applied by topical coating twice a day until the analysis was completed. *p ≤ 0.05, ^#^power > 0.8.

Notably, mFGF8b also could enhance the rate of wound repair (**Figure** **9**A). HE staining illustrated that the application of mFGF8b promoted both epithelial cell migration and regeneration (Figure 9B,C). Masson's trichrome staining revealed that mice treated with mFGF8b exhibited higher collagen levels and improved tissue structure compared to the control (Figure 9D). To further explore whether mFGF8b facilitates wound repair via glycolysis, we inhibited glycolysis at the wound sites with 2‐DG. As shown in Figure 9E, mice treated with both 2‐DG and mFGF8b exhibited decreased rates of epithelial cell migration, regeneration, and collagen content compared to those treated with mFGF8b alone. These findings indicate that mFGF8b also has direct antibacterial activity and supports re‐epithelialization by enhancing glycolysis.

## 3 Discussion

In tetrapods, alternative splicing of the *FGF8* gene results in multiple variants. For instance, mouse FGF8 has 8 variants (a, b, c, d, e, f, g, h), while human FGF8 has 4 (a, b, e, f).^[^ ^26^ ^]^ In contrast, teleosts lack splice variants but possess two distinct genes, *FGF8a* and *FGF8b*. Evolutionary analysis has shown that teleost FGF8a, but not FGF8b, has higher homolog with tetrapod FGF8s. However, the expression patterns of teleost *FGF8a* is different from that of tetrapod *FGF8s*. In tetrapods, *FGF8* is highly expressed in the reproductive and urogenital tracts,^[^ ^38^ ^]^ and its expression increases during embryonic development and organogenesis.^[^ ^39^, ^40^ ^]^ In this study, we found that *gcFGF8a* is highly expressed in mucosal immune organs in response to bacterial infections and shows elevated expression at skin ulceration sites, suggesting the association of gcFGF8a with bacterial infections.

Given the unique evolutionary position and habitat, teleosts rely heavily on immune effectors within mucosal immune organs to maintain health.^[^ ^41^ ^]^ Therefore, the potential role of gcFGF8a in mucosal immunity sparked our interest. Our findings revealed that gcFGF8a possesses direct and broad antibacterial activity, serving as an AMP to help grass carp resist *A. hydrophila* infection. Although AMPs are promising antibacterial drugs, their cytotoxicity is a major challenge.^[^ ^42^, ^43^ ^]^ Unlike eukaryotic cell membranes, bacterial cell membranes contain negatively charged acidic lipids, which allows cationic AMPs to preferentially bind to bacterial membranes.^[^ ^27^ ^]^ Our study demonstrated that gcFGF8a also exploits this distinction, recognizing bacteria and exhibiting lower eukaryotic cytotoxicity. Like most cationic AMPs, gcFGF8a has a cationic amphiphilic feature, forming the basis of its direct antibacterial activity.^[^ ^25^ ^]^ Because the positively charged surface of gcFGF8a facilitates its attraction to negatively charged components of the bacterial surface, such as LPS, LTA, and PGN.^[^ ^44^, ^45^ ^]^ Like other cationic AMPs,^[^ ^46^, ^47^, ^48^ ^]^ gcFGF8a can kill both Gram‐negative and Gram‐positive bacteria by disrupting cell membranes, which was supported by the membrane permeability test, TEM and SEM observations.

The expression of *gcFGF8a* significantly increased at skin ulceration sites, suggesting its involvement in wound healing and ulcer regeneration. Furthermore, its low expression during early injury stages followed by a surge during the repair phase highlights its crucial role in the healing process. This expression pattern resembles that of other growth factors, such as EGF, PDGF, and TGF‐β, which contribute to skin homeostasis.^[^ ^49^, ^50^ ^]^ Previous studies have established that wound healing involves a coordinated response of multiple cell types, with leukocytes initially infiltrating the wound to combat infection and prevent blood loss, followed by fibroblasts proliferating to deposit collagen and regenerate tissue.^[^ ^1^ ^]^ Our study aligns with this paradigm, demonstrating that gcFGF8a enhances leukocyte recruitment at day 3 post‐injury and collagen deposition at day 7. Additionally, leukocytes serve as the first line of defense against pathogens and regulate subsequent stages of wound repair by releasing chemotactic factors, cytokines (such as TNF‐α, IL‐1, and IL‐6), and growth factors (including TGF‐α/β and IGF‐1).^[^ ^51^, ^52^, ^53^ ^]^ Subsequently, in the gcFGF8a treated group, fibroblasts enriched at the wound site, promoting collagen deposition and initiating tissue formation and remodeling.^[^ ^54^ ^]^ Moreover, gcFGF8a reduced the bacterial load of *Aeromonas* at the wound site, aiding in defense against bacterial infection. Collectively, gcFGF8a helps prevent the disruption of wound healing equilibrium by bacterial infection, thus facilitates the repair process.^[^ ^2^, ^3^, ^4^, ^5^ ^]^

Our study elucidated a novel signaling axis linking bacterial infection, Wnt‐dependent gcFGF8a induction, and FGFR4‐mediated mTORC1‐HIF1α activation in teleost wound repair. Bacterial infection triggers Wnt signaling and upregulates gcFGF8a, which is consistent with previous reports that Wnt/β‐catenin pathway activation increases FGF8 expression.^[^ ^55^ ^]^ Our findings also revealed that acute skin injuries induce a hypoxic microenvironment in teleosts, marked by increased *HIF1α* expression.^[^ ^34^ ^]^ Additionally, during wound healing, the expression levels of *HIF1α* and *gcFGF8a* synchronously increased, suggesting a potential association. Unlike VEGF, which is activated by mTOR‐HIF1α, gcFGF8a binds FGFR4 and activates mTOR through ERK and AKT kinases, promoting HIF1α secretion by epithelial cells.^[^ ^56^ ^]^ As a key regulator of hypoxic signaling and glycolysis, HIF‐1α plays a crucial role in tissue repair processes.^[^ ^57^, ^58^ ^]^ Collectively, our study showed that gcFGF8a upregulates HIF1α, accelerating glycolysis to provide energy for tissue survival and repair during healing.^[^ ^37^ ^]^

Given the direct antibacterial properties of gcFGF8a and its ability to enhance wound healing through activating glycolysis, we are curious whether the functions of FGF8 are conserved in vertebrates. While FGF8 exhibits alternative splicing in tetrapods, generating various variants, FGF8b stands out for its widespread tissue distribution and higher affinity for FGFRs.^[^ ^59^ ^]^ Sequence analysis shows that among the mouse FGF8 isoforms, mFGF8b exhibits the closest resemblance to teleost FGF8a. Moreover, like gcFGF8a, mFGF8b also exhibits a cationic amphiphilic structure. Therefore, we purified mFGF8b and found that it also has direct antibacterial activity and facilitates wound healing by activating glycolysis. These results indicate that the antibacterial and wound healing function of FGF8 are conserved in vertebrates.

**Figure 10:**
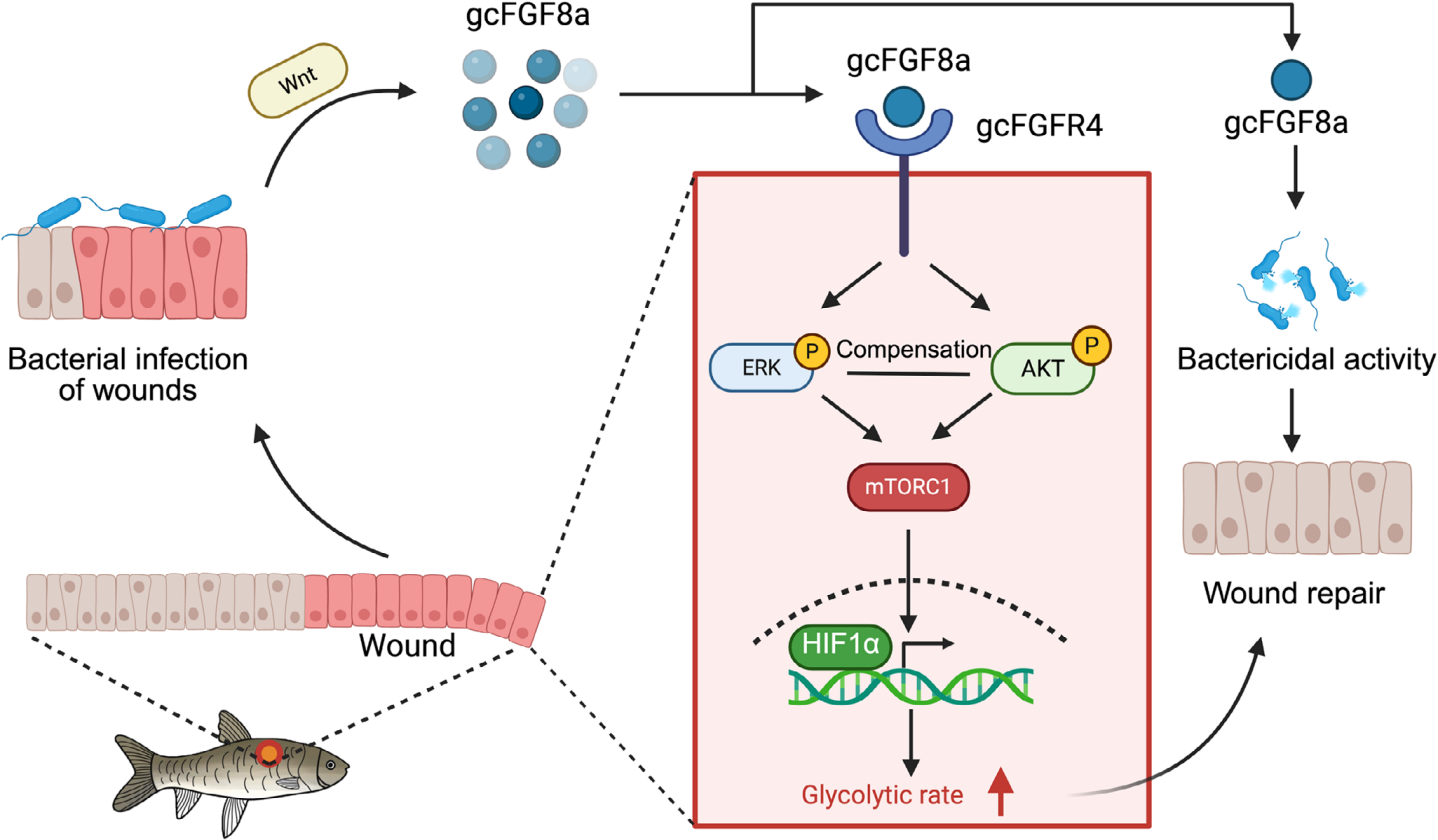
Schematic diagram illustrating the dual mechanisms of gcFGF8a in wound repair. Wound infections induce gcFGF8a expression, which subsequently executes direct antimicrobial activity to suppress local infection while simultaneously activating the FGFR4‐mediated ERK/AKT‐mTOR signaling cascade, thereby upregulating HIF1α and enhancing glycolysis. These coordinated actions synergistically promote tissue repair by eliminating pathogens and enhancing glycolysis.

In summary, we have uncovered a new AMP, FGF8, which plays a pivotal role in skin wound healing by directly inhibiting bacteria and activating glycolysis in both grass carp and mouse (**Figure** **10**). To our knowledge, FGF8 is currently the only member of the FGF family with antibacterial activity, offering potential for skin wound treatment. These findings significantly advance our understanding of FGFs and highlight the application of FGF8 for skin wound healing.

## 4 Conclusion

The high expression of fibroblast growth factor 8 (FGF8) in teleost mucosal tissues suggests a novel role of FGF8 in mucosal immunity. Through the integration of molecular techniques and both in vitro and in vivo experimental models, this study delineates the dual roles of FGF8 in antimicrobial defense and metabolic regulation. Specifically, FGF8 exerts bactericidal activity by disrupting bacterial membranes, while concurrently promoting wound repair by enhancing glycolysis via the mTOR‐HIF1α pathway. Importantly, these functional properties are conserved across vertebrates, underscoring the evolutionary significance of FGF8. As the only FGF family member discovered to possess both antibacterial and wound repair roles, FGF8 offers new insights into its biological importance and potential for developing innovative wound healing therapies.

## 5 Experimental Section

### Animals

Natural diseased and healthy grass carp (20 ± 5 g) were obtained from Tuanfeng Fish Breeding Base (Tuanfeng, China). BALB/C SPF mice were purchased from the Experimental Animal Center of Huazhong Agricultural University. All the experimental protocols were approved by the Animal Ethics Committee of Huazhong Agricultural University (HZAUFI‐2023‐0005, HZAUMO‐2024‐0310).

### Drug Treatment—2‐Deoxy‐D‐Glucose (2‐DG) and BAY872243

In the in vitro scratch wound assay, 2‐Deoxy‐D‐glucose (2‐DG, MCE), an inhibitor of glycolysis, and BAY872243 (MCE), an inhibitor of HIF1α, was added to EPCs to 40 and 2 µM, respectively, 1 h before the scratch test. The cells were cultured at 28 °C and the photographs were taken at 0 and 24 h, respectively. In the in vivo wound healing experiment of grass carp, 100 µL PBS containing 15 ng 2‐DG was injected into the muscle near the wound site. In mice, the same concentration of 2‐DG was topically applied around the wound twice a day until the analysis was completed.

### Drug Treatment—Rapamycin, SCH772984, MK‐2206, and BLU‐554

In the in vitro gcFGF8a stimulation assay, EPCs were treated with 60 nM rapamycin (MCE), an inhibitor of mammalian target of rapamycin (mTOR), 1 µM SCH772984 (MCE), an inhibitor of extracellular regulated protein kinase (ERK), 5 µM MK‐2206 (MCE), an inhibitor of Protein Kinase B (AKT), or 5 nM BLU‐554 (MCE), an inhibitor of FGFR4 for 3 h before sampling. The inhibitor concentrations were selected based on the concentrations recommended in the product manual.

### Drug Treatment—BAY11‐7082, Tofacitinib, LY29402, SB431542, and IWR‐1

In the *A. hydrophila* infection assay, cells were pretreated with specific signaling pathway inhibitors for 3 h prior to infection: 1 µM BAY11‐7082 (MCE), an inhibitor of NF‐κB, 1 µM Tofacitinib (MCE), an inhibitor of JAK, 20 µM LY294002 (MCE), an inhibitor of PI3K, 10 µM SB431542 (MCE), an inhibitor of TGF‐β, or 5 µm IWR‐1 (MCE), an inhibitor of Wnt. The inhibitor concentrations were selected based on published studies and preliminary optimization to ensure effective pathway inhibition while maintaining cell viability. Following inhibitor pretreatment, cells were infected with *A. hydrophila* at a multiplicity of infection (MOI) of 10 for specified time points before subsequent analysis.

### Sequence Analysis and Structure Modeling

The phylogenetic tree was constructed based on protein sequences of FGF8 using the minimum‐evolution method with 1000 bootstrap replicates by MEGA software (version 7.0). Multiple sequence alignment was performed using the ClustalW program (https://www.genome.jp/tools‐bin/clustalw) and visualized using the ESPript program (https://espript.ibcp.fr/ESPript/cgi‐bin/ESPript.cgi). Molecular weight (MW), isoelectric point, and hydrophobic content of FGF8s were calculated using DNASTAR software (Madison). The determination of FGF8s’ (R+K)% and Pho% (ratio of hydrophobic amino acid residues) was carried out by utilizing the online AMP database (http://aps.unmc.edu/AP).^[^ ^60^ ^]^ The AlphaFold 2.0 software was employed to model the tertiary structures of FGF8s and the structures were visualized by an online software (http://gsds.gao‐lab.org/).

### Expression and Purification of Recombinant FGF8s in *Escherichia coli*

FGF8s of grass carp (gcFGF8a) and mouse (mFGF8b) were expressed in *E. coli* BL21(DE3) Rosetta cells as a fusion protein containing a C‐terminal 6×His‐tag. Briefly, the expression of recombinant FGF8s were induced with 0.75 mM isopropyl β‐D‐1‐thiogalactopyranoside at 37 °C for 6 h. Then, the inclusion bodies were isolated, solubilized in 6 M guanidine hydrochloride, and purified by Ni‐NTA Resin (GE Healthcare). Refolding was performed by dilution in refolding buffer (20 mM Tris, 400 mM arginine, 2 mM EDTA, 5 mM reduced glutathione, 0.5 mM oxidized glutathione, and 0.1 mM PMSF, pH 8.0). After that, recombinant FGF8s were dialyzed in Tris buffer (20 mM, pH 7.4) and then stored at −80 °C until use. The purity of the recombinant proteins was identified using SDS‐polyacrylamide gel electrophoresis (SDS‐PAGE). The contamination of endotoxin in recombinant FGF8s was detected to be negligible (<0.04 EU/mg) using an endotoxin detection kit (GenScript).

### Expression Patterns of gcFGF8a Genes

The expression of *gcFGF8a* in ulcerated fish was analyzed after extracting RNA from naturally diseased and healthy fish skin tissues. To detect the tissue expression pattern of *gcFGF8a*, total RNA was isolated from the head‐kidney, body‐kidney, spleen, blood, gut, skin, gill, liver, muscle, heart, eye, and brain of euthanized grass carp. Meanwhile, grass carp were intraperitoneally injected with *Aeromonas hydrophila* (1 × 10^7^ CFU mL ^−1^ , 100 µL per fish), and total RNA was isolated from the gut, gill, skin, spleen, head‐kidney, and blood of the fish at 0, 12, 24, 36, and 72 h post‐injection. RNA was reverse transcribed into cDNA using Hiscript III Reverse Transcriptase (Vazyme). Quantitative real‐time PCR (RT‐qPCR) was performed using TSINGKE® Master qPCR Mix (Tsingke Biotech) in a CFX ConnectTM Real‐Time System (Bio‐Rad) to analyze the expression levels of *gcFGF8a*. The primers used are listed in Table S1 (Supporting Information). Using 18S rRNA gene as an internal control, the expression levels of *gcFGF8a* under normal and challenged conditions were analyzed by 2^−ΔΔCT^ method.^[^ ^61^ ^]^

### Bacterial Infection in Grass Carp

To evaluate the therapeutic effect of gcFGF8a on bacterial infection, an infection model was established by intraperitoneally injecting *A. hydrophila* (2×10^6^ CFU/fish) in grass carp as previously described.^[^ ^62^ ^]^ After 12 h, fish were injected intraperitoneally with recombinant gcFGF8a (1 µg g^−1^) or an equal volume of PBS.^[^ ^46^ ^]^ The survival rate was monitored twice a day over 7 days. On day 3 post‐infection, some individuals were anesthetized with 0.02% 3‐Aminobenzoic acid ethyl ester methanesulfonate (MS‐222), and the skin tissues were sampled. After weighing, the tissue was homogenized in PBS (0.1 g mL^−1^) using a tissue grinder (Sigma). The tissue homogenates were diluted and plated onto TSA plates (Hope Bio) to count the skin bacterial load. For immersion infection, grass carp were immersed in *A. hydrophila* (5 × 10^6^ CFU mL^−1^) for 20 min and subsequently transferred to the culture environment.^[^ ^63^ ^]^

### Antibacterial Activity Assay

Antibacterial activity of gcFGF8a against the following bacteria was determined by CFU assay: *E. coli* ATCC25922, *A. hydrophila* ZYAH72, *Yersiniavan ruckeri* ATCC 29473, *Pseudomonas fluorescens* ATCC17386, *Staphylococcus aureus* ATCC25923, *Streptococcus agalactiae* ATCC13813, and *Micrococcus luteus* ATCC10240.^[^ ^46^ ^]^ Briefly, the bacteria in logarithmic growth phase (OD_600_ = 0.5) were diluted to 5 × 10^5^ CFU mL^−1^ using Tris buffer (20 mM, pH 7.4). Then, 100 µL of bacteria was mixed with an equal volume of five‐fold serial dilutions of gcFGF8a protein, and incubated at 37 °C (28 °C for *A. hydrophila*) for 3 h. After that, 20 µL of the treated bacteria was plated onto TSA agar plates and incubated at 37 °C (28 °C for *A. hydrophila*) for 12 h. Finally, the CFUs were counted.

### Cytotoxicity Assay

SYTO9/PI staining (LIVE/DEAD) was used to detect the cytotoxicity of FGF8s to grass carp head kidney leukocytes (HKLs). HKLs were enriched using (34%/51%) Percoll density gradient centrifugation as previously described.^[^ ^64^ ^]^ Then, HKLs were seeded in 96‐well plate at a density of 10^4^ cells per well and treated with 50 µM FGF8s or LL37 at 28 °C for 12 h. Subsequently, dual staining with SYTO9 (2 µM) and PI (10 µM) was performed, and the detection was carried out using a flow cytometer FACSVerseTM (BD Biosciences). The cytotoxicity of FGF8s and LL‐37 to grass carp erythrocytes was determined as described previously.^[^ ^65^ ^]^ Briefly, grass carp erythrocytes were prepared as an 8% (v/v) suspension using the previously described method.^[^ ^62^ ^]^ Subsequently, the erythrocytes were incubated with serially diluted FGF8s or LL‐37 for 2 h. The hemolysis percentage was determined by measuring the OD_405_ of the supernatant after centrifugation at 500 × *g* for 10 min.

### Lipid‐Targeting and Liposome Disruption Assays

Membrane lipid strip (Echelon) was used to detect the binding properties of gcFGF8a to different lipids following the manufacturer's instructions. Briefly, the lipid strip was blocked with 5% BSA at room temperature for 2 h. Subsequently, gcFGF8a was diluted to 1 µg mL^−1^ in 5% BSA and incubated with the lipid strips overnight at 4 °C. After incubation, the strips were washed three times and incubated with mouse anti‐His monoclonal antibody (mAb; ABclonal) at room temperature for 2 h. Following three additional washes, the strips were incubated with HRP‐conjugated goat anti‐mouse IgG (ABclonal) at room temperature for 45 min. Then, ECL reagent (Biosharp) was added and the signal was detected using the ChemiDoc imaging system (Bio‐Rad).

Liposomes were preparation following established protocols with minor changes.^[^ ^66^, ^67^ ^]^ Briefly, 1‐palmitoyl‐2‐oleoyl‐*sn*‐glycero‐3‐phosphocholine (PC), 1,2‐dioleoyl‐*sn*‐glycero‐3‐phospho‐L‐serine (PS), and PC:PS mixtures (85% PC and 15% PS) were dissolved in chloroform, dried under nitrogen, and hydrated with 100 mM 5(6)‐carboxy‐fluorescein (CF, Sigma‐Aldrich). After seven freeze‐thaw cycles, the liposomes were purified using desalting column, and then diluted to 100 mM in assay buffer (10 mM Tris, pH 7.5). Fluorescence measurements (excitation, 490 nm; emission, 516 nm) were performed at 200 s post‐dilution, then samples were incubated with gcFGF8a (1 µg mL^−1^) for 5 min before recording final fluorescence values. Total dye release was determined by adding 1% *n*‐octyl glucoside (OG). All readings were normalized to initial fluorescence and maximum OG‐induced release.

### Binding Assay of FGF8s to Bacteria and PAMPs

Western blot was employed to assess the binding activity of FGF8s to various bacteria.^[^ ^46^ ^]^ In brief, bacteria were incubated with FGF8s (0.1 µM) for 1 h, followed by three washes with Tris buffer (20 mM, pH 7.0). Subsequently, bacteria were boiled, separated by SDS‐PAGE, transferred onto a nitrocellulose membrane (Bio‐Rad), incubated with mouse anti‐His mAb (ABclonal) and HRP‐conjugated goat anti‐mouse IgG (ABclonal). Then, ECL reagent (Biosharp) was added and the signal was detected using the ChemiDoc imaging system (Bio‐Rad). Additionally, the binding activity of different concentrations of FGF8s to *E. coli* and *S. aureus* was investigated.

The binding activity of gcFGF8a to PAMPs was detected using bio‐layer interferometry (BLI) and enzyme‐linked immunosorbent assays (ELISA) as previously described.^[^ ^46^, ^68^ ^]^ Briefly, BLI experiments were performed using the BLItz system (ForteBio Octet RED96e) with Ni‐NTA sensors to immobilize His₆‐tagged gcFGF8a. Binding affinities of gcFGF8a to LPS, PGN, and LTA (1 nM in 20 mM Tris‐HCl, pH 7.4) were measured at 28 °C, and KD values were calculated using a 1:1 binding model. For ELISA, 8 µg of LPS (*E. coli*), PGN (*M. luteus*), or LTA (*S. aureus*) were coated on ELISA plate overnight at 4 °C. Subsequently, the plate was blocked with 5% BSA (200 µL per well) at 37 °C for 3 h. After washed, 2‐fold serial dilutions of gcFGF8a protein were added and incubated for 1 h. Thereafter, mouse anti‐His monoclonal antibody (ABclonal) and HRP‐conjugated goat anti‐mouse IgG (ABclonal) were used as primary and secondary antibodies, respectively. Finally, the plate was washed, and TMB solution (YEASEN) was incubated for 5–30 min. After color development, absorbance at 405 nm was read using a plate reader (Bio‐Tek).

### Propidium Iodide (PI) Uptake Assay

The PI uptake assay was utilized to assess the membrane permeability of bacteria treated by FGF8s.^[^ ^62^ ^]^ *E. coli* or *S. aureus* at a concentration of 10^6^ CFU mL^−1^ were treated with varying concentrations of FGFs (0, 1, 5, 10 µM) at 37 °C for 1 h. Subsequently, bacteria were collected and incubated in PBS containing 10 µg mL^−1^ PI for 30 min. The influx of PI was measured using a flow cytometer FACSVerse^TM^ (BD Biosciences). In this experiment, LL37 was employed as a positive control.

### Transmission Electron Microscopy (TEM) and Scanning Electron Microscopy (SEM) Imaging

The morphological and microstructural changes in bacteria treated with FGF8s were observed through TEM and SEM as previously described.^[^ ^69^ ^]^ Briefly, bacterial (5 × 10^7^ CFU) were treated with FGF8s (5 µM) in 20 mM Tris (pH 7.0) at 37 °C for 1 h. Subsequently, the samples were fixed overnight at 4 °C using 2.5% glutaraldehyde. For TEM, the treated bacteria were fixed, embedded, sliced, and observed using TEM (Hitachi H‐76500). In SEM, the treated bacteria were fixed, dehydrated, vacuum‐dried, sputter‐coated with gold, and observed using field emission SEM (Hitachi S‐4800).

### Skin Wound Model

In grass carp (*n* = 50), two standard circular skin wounds (diameter 4 mm) were created on the dorsal side using a biopsy punch. Following the injury, samples from the wounds were collected on days 0, 1, 2, and 3 to assess bacterial load and tissue change. For bacterial load, after weighing, tissue homogenates were prepared using a tissue grinder (Sigma) in PBS (0.1 g mL^−1^). The homogenized tissues were then diluted and plated on TSA agar plates (Hope Bio) for colony counting. Additionally, in wound samples, 60 colonies were randomly selected for 16S rDNA sequencing to determine the bacterial species. For tissue staining, tissues were fixed in 10% paraformaldehyde and subjected to routine hematoxylin and eosin (HE) staining to observe tissue damage. Masson's trichrome staining was also performed to assess the growth of collagen fibers. Additionally, wound tissues were collected at 0, 1, 2, 3, 7, 14 days post‐trauma and detected the expression of *gcFGF8a* and *HIF1α* by RT‐qPCR. Subsequently, to evaluate the wound healing effects of gcFGF8a, grass carp were randomly divided into two groups. One group (*n* = 20) was soaked with gcFGF8a protein (3 mg L^−1^) twice daily after the skin trauma for 7 days, while the other group (*n* = 20) was soaked with an equal volume of PBS. The tissue homogenate was serially diluted and plated onto *Aeromonas* spp. selective plates (Rimler–Shotts medium; Hope Bio) to count the number of *Aeromonas* spp. The HE staining, and Masson's trichrome staining of the wound sites were performed using the method described above. Collagen fiber growth in the Masson's trichrome‐stained tissue sections was quantified using Image Pro Plus software.

In mice, following previous studies, four circular skin wounds (diameter 4 mm) were created on the dorsal side using a biopsy punch after shaving the back fur.^[^ ^70^, ^71^ ^]^ Mice were randomly divided into two groups. One group (*n* = 15) received topical coating of mouse FGF8b (mFGF8b) protein (3 mg L^−1^) twice a day after trauma for 14 days, while the other group (*n* = 15) received an equal volume of PBS. Using the method described above, the HE staining, and Masson's trichrome staining of the wound sites were performed. Collagen fiber growth in the Masson's trichrome‐stained tissue sections was quantified using Image Pro Plus software.

### Cell Proliferation Assay

In this study, the influence of gcFGF8a on the proliferation of EPCs was investigated using the BeyoClick™ EdU‐488 Assay Kit (Beyotime Biotechnology). EPCs (1 × 10^5^ cells) were cultured in a 96‐well plate at 28 °C for 24 h. Subsequently, different concentrations of gcFGF8a, along with 1 µm 5‐ethynyl‐2’‐deoxyuridine (EdU), were used to stimulate EPCs for 24 h. Stimulated cells were then collected, and the proliferation of EPCs was measured using flow cytometry according to the manufacturer's instructions.

### Scratch Wound Assay

EPCs were seeded in a 12‐well plate at a density of 5 × 10^5^ cells per well and cultured at 28 °C for 24 h to achieve a confluent monolayer. Linear scratches were generated using a 200 µL sterile pipette tip, followed by washing cell debris with PBS. Subsequently, 1.5 mL serum‐free medium 199 containing 0, 10, 100, or 1000 ng mL^−1^ of gcFGF8a was added to each well. The plates were incubated at 28 °C, and photographs were taken at 0 and 24 h. In the inhibitor experiment, 2‐DG (40 µM) and BAY872243 (2 µM) were added 1 h before the scratch assay. The distance of closure for each scratch and the migration rates were quantified using Image Pro Plus software.

### Cell Migration Assay

Cell migration was evaluated using a Transwell system (8‐µm pore polycarbonate membrane, Corning) where 4 × 10⁴ EPCs suspended in 200 µL serum‐free M199 were seeded into the upper chamber, while the lower chamber contained 700 µL M199 supplemented with varying concentrations of gcFGF8a (10, 100, 1000 ng mL^−1^). After 6‐h incubation at 28 °C, non‐migratory cells were removed using cotton swabs, and migrated cells on the lower membrane surface were fixed with 4% paraformaldehyde, stained with 0.1% crystal violet, and quantified using ImageJ software by analyzing three random fields per membrane.

### Detection of Proteins by Western Blot

EPCs were collected and lysed using RIPA lysis buffer (Biosharp), and the protein concentration was determined using a BCA kit (Beyotime). Subsequently, the protein lysate was incubated at 100 °C for 10 min after adding loading buffer. Equal amounts of protein were separated by SDS‐PAGE and transferred onto a nitrocellulose membrane (Bio‐Rad). The membrane was then blocked with 5% nonfat dried milk in TBST and incubated with the primary antibodies: rabbit anti‐HIF1α mAb (ABclonal), rabbit anti‐P‐mTOR pAbs (ABclonal), rabbit anti‐P‐ERK pAbs (Proteintech), rabbit anti‐P‐AKT pAbs, (ABclonal) and rabbit anti‐actin mAb (ABclonal). Following that, the membrane was incubated with HRP‐conjugated goat anti‐rabbit IgG pAbs (ABclonal), and protein bands were visualized using Clarity Western ECL substrate (Bio‐Rad) and analyzed with the Amersham Imager 600 (GE Healthcare).

### RNA Sequencing

EPCs were cultured overnight in six‐well plates and treated with gcFGF8a (100 ng mL^−1^) or equal volume of PBS for 12 h prior to RNA isolation using Trizol Reagent (Invitrogen). After quality assessment by NanoDrop spectrophotometry (Thermo Scientific), RNA‐seq libraries were constructed using 3 µg RNA according to Illumina protocols: mRNA was captured with poly‐T beads, fragmented with divalent cations, and reverse‐transcribed with random hexamers, and then the second‐strand cDNA was synthesized. Blunt‐ended fragments were adenylated, ligated to Illumina adapters, size‐selected (400–500 bp) with AMPure XP beads (Beckman Coulter), and amplified (15 cycles). Libraries were sequenced on the NovaSeq 6000 sequencing system (Illumina). Bioinformatic analysis of the sequences was performed as follows: alignment to genome via STAR (v2.4.2a), quantification with FeatureCounts (v1.4.6), normalization using TMM (EdgeR) and quantile methods, and pathway enrichment analysis of genes with |Log₂FC| ≥ 0.5 via Ingenuity Pathway Analysis (IPA).

### Pull‐Down Assay

His‐tagged gcFGF8a protein (5 µg) was immobilized on Ni‐NTA agarose beads and incubated with whole‐cell lysates of EPCs (500 µg, prepared using RIPA lysis buffer) at 4 °C for 2 h with constant mixing. Empty Ni‐NTA agarose beads were included as a negative control. Following incubation, the beads were washed several times with RIPA buffer to remove non‐specifically bound proteins. The bound proteins were then eluted and analyzed by Western blot using rabbit anti‐FGFR4 mAb (ABclonal) to detect FGFR4. Subsequent to Western blot analysis, the proteins were resolved by SDS‐PAGE, and the gel was cut into two pieces at approximately 70 kDa. Both the upper and lower gel pieces, containing proteins larger and smaller than 70 kDa respectively, were excised and analyzed by mass spectrometry to identify the interacting proteins.

### Extracellular Acidification Rate Analysis

Extracellular acidification rate (ECAR) analysis was performed using the Agilent Seahorse Xfe24 Analyzer with Seahorse XF Glycolytic Rate Assay Kit. Briefly, EPCs were seeded in Agilent Seahorse 24‐well XF cell culture microplates at a density of 4 × 10^4^ cells per well in 150 µL of growth medium. After incubated for 12 h in a 28 °C humidified incubator with 5% CO2, the cells were stimulated with gcFGF8a (1000 ng mL^−1^) for 24 h. The Seahorse XF Sensor Cartridge was hydrated in calibrant solution at 28 °C overnight before use. The rotenone/antimycin A (0.5 µM) and 2‐DG (50 mM) were sequentially injected, and the proton efflux rate was detected.

### Measurement of Enzyme Activity and Lactate Secretion

The enzyme activities of lactate dehydrogenase (LDH), hexokinase (HK), and phosphofructokinase (PFK) in cells were detected using the corresponding assay kits from Nanjing Jiancheng Bioengineering Institute. The enzyme activities of fructose‐bisphosphate aldolase (FBA) and pyruvate kinase (PK) were measured using the respective assay kits from Solarbio. For lactate secretion assay, the supernatant from the cell culture was harvested, centrifuged at 1000 × *g* to pellet debris. The lactate content was measured using the microplate‐based CheKine™ Lactate Assay Kit (Abbkine) according to the manufacturer's instructions.

### Statistical Analysis

Data analysis was performed using Student's *t*‐test (two‐tailed, unpaired) with GraphPad Prism 7.0 software (GraphPad). Statistical significance was defined as *p* ≤ 0.05. The SPSSAU program was utilized to perform power analysis, with statistical significance defined as Power > 0.8.

### Ethical Statement

All the experimental protocols were approved by the Animal Ethics Committee of Huazhong Agricultural University (HZAUFI‐2023‐0005, HZAUMO‐2024‐0310).

## Conflict of Interest

The authors declare no conflict of interest.

## Author Contributions

Y.‐Z.H., Y.‐A.Z., and X.‐J.Z. performed conceptualization and design. Y.‐Z.H., T.W., C.‐S.W., J.‐W., and X.‐Q.H. performed the acquisition, analysis, or interpretation of data and experiments. Y.‐Z.H. drafted the manuscript. Y.‐A.Z. and X.‐J.Z. performed critical revision of the manuscript for important intellectual content. Y.‐Z.H., C.‐S.W., J.‐W., and X.‐Q.H. performed the statistical analysis. Y.‐A.Z. and X.‐J.Z. acquired funding. Y.‐A.Z. and X.‐J.Z. did the supervision.

## Supporting information



Supporting Information

## Data Availability

The data that support the findings of this study are available from the corresponding author upon reasonable request.
