# Supplementary material for: Novel Antimicrobial Protein Fibroblast Growth Factor 8 Accelerates Skin Wound Healing via Directly Inhibiting Bacteria and Activating Glycolysis
Source: Adv Sci (Weinh). 2025 Jun 30;12(36):e00388. doi: 10.1002/advs.202500388 (PMC12463102; doi:10.1002/advs.202500388)
Supplement: Supplementary file 1 — Supporting Information [file ADVS-12-e00388-s001.doc]

Supporting Information

Novel antimicrobial protein fibroblast growth factor 8 accelerates skin wound healing via directly inhibiting bacteria and activating glycolysis

*Ya-Zhen Hu, Ting Wang, Chang-Song Wu, Jie Wang, Xue-Qing Han, Yong-An Zhang,* and Xu-Jie Zhang**

## Supplemental Table 1. Primers used in this study.

| Species | Gene name | Application | Primer sequence (5'–3') |
| --- | --- | --- | --- |
| grass carp | FGF8a | plasmid construction | **CGG***ccatgg*(*Nco* Ⅰ)ACTCCCCGCCTAATTTTACACAG |
|  |  |  | **CGG***ggatcc*(*Bam*H Ⅰ)TCAATGATGATGATGATGATGACGCTCTCCTGAGTAGCG |
| grass carp | FGF8a | qRT-PCR | GCTCCAAAACCAGGCAACAC |
|  |  |  | TGAGTAGCGGGTGCGTTTAG |
| grass carp | HIF1α | qRT-PCR | CAGTGCATTGTGTGCGTCAA |
|  |  |  | GCACTCCATGGGACACTTCA |
| grass carp | Aldoa | qRT-PCR | ACACTCAACCTGAACGCCAT |
|  |  |  | TAGGCATGGTTGGCCACAAA |
| grass carp | Ldha | qRT-PCR | CAAACCTGGACTCTGCTCGT |
|  |  |  | TTGTGGACGCTCTTCCAGTC |
| grass carp | Pkm | qRT-PCR | CCGTCGAGTCGTCCTTCAAA |
|  |  |  | GACGGGCCACCTGAACATTA |
| grass carp | Hk | qRT-PCR | GAGTTCCGGCTGACCAAAGA |
|  |  |  | TCTGGGGTACTCCGCACATA |
| grass carp | Pfk | qRT-PCR | CAGTTACGGGGCAGGAGTTT |
|  |  |  | ACGACACTTCATTCACCGCT |
| grass carp | 18S rRNA | qRT-PCR | ATTTCCGACACGGAGAGG |
|  |  |  | CATGGGTTTAGGATACGCTC |
| grass carp | β-actin | qRT-PCR | GCCCCACCTGAGCGTAAATA |
|  |  |  | GAGTCGGCGTGAAGTGGTAA |
| common carp | HIF1α | qRT-PCR | GAGCGGAGGACTTCCCTTTC |
|  |  |  | CCTGGGAGCTAAAAGGGGTC |
| common carp | Aldoa | qRT-PCR | GCATCAATGCGGAGAACACC |
|  |  |  | AACTACCATGCCCCTCTCCT |
| common carp | Ldha | qRT-PCR | CGTCCAGAGGAACGTCAACA |
|  |  |  | CGAGCAGAGTCCAGGTTTGT |
| common carp | Pkm | qRT-PCR | AGAAGGGTGTCAATCTGCCG |
|  |  |  | GAGCACCTTACGCACCTCAT |
| common carp | Hk | qRT-PCR | TCTGCGGGTGAAAGTGTCTC |
|  |  |  | CGCCTCATCCAGTTTGGAGT |
| common carp | Pfk | qRT-PCR | GATCGGACTTCAGCATCGGT |
|  |  |  | CCTGCCATGGTTGCCAGATA |
| common carp | β-actin | qRT-PCR | GCTATGTGGCTCTTGACTTCGA |
|  |  |  | CCGTCAGGCAGCTCATAGCT |

Note: The nucleotides in bold represent protective bases, lowercase letters indicate the positions of the restriction enzyme sites, and the underlining stands for the His tags.

**
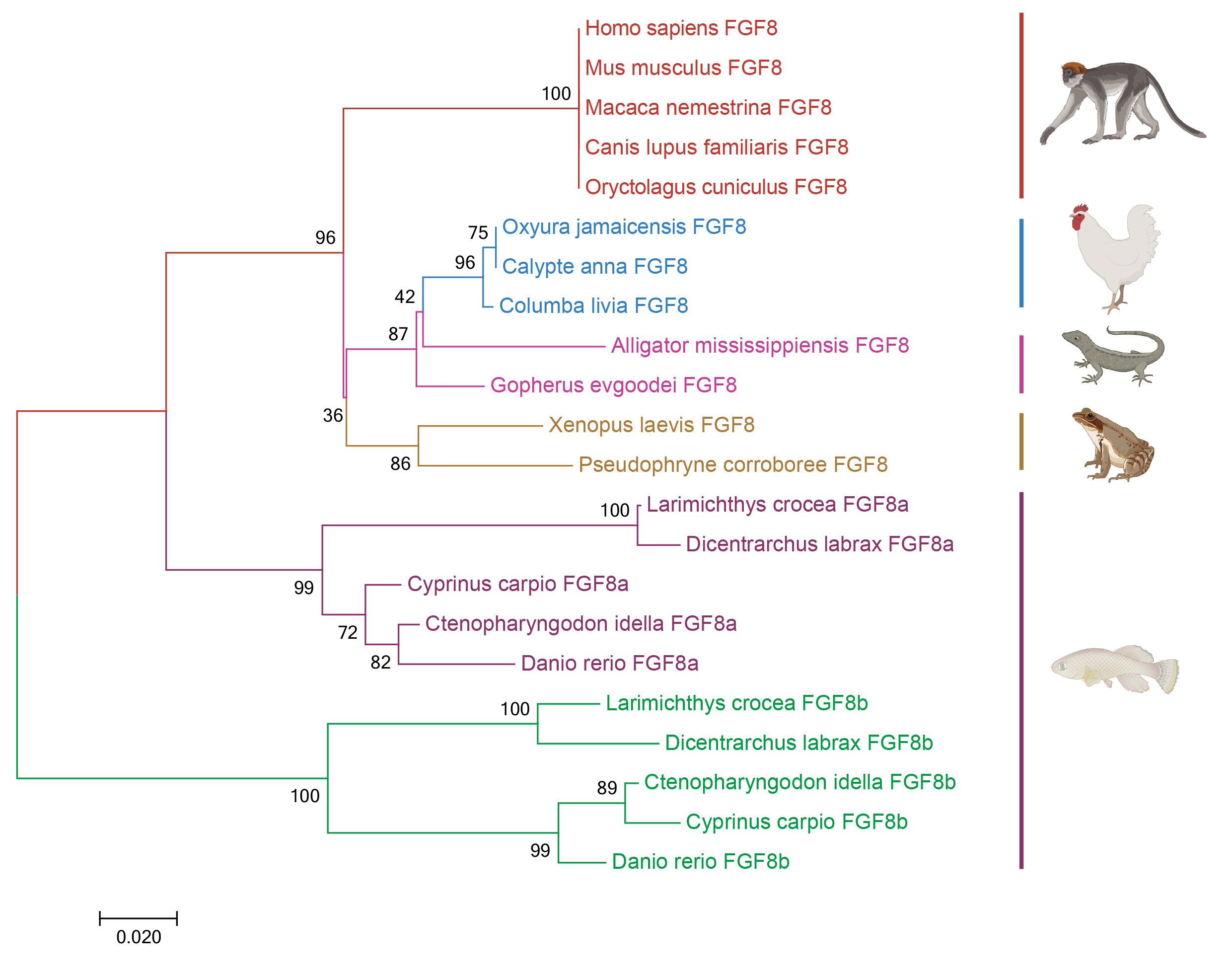
**

## Supplemental Fig. 1 Phylogenetic relationship between the teleost FGF8s and tetrapod FGF8s. Based on the FGF8 protein sequences, a phylogenetic tree was constructed by the minimal evolution method with 1000 times of initiation using MEGA software. The GenBank accession numbers of FGF8 used are as follows: *Homo sapiens* *FGF8*, U46212.1; *Mus musculus FGF8*, NM_001166362.2; *Macaca nemestrina FGF8*, XM_024791752.1; *Canis lupus familiaris* *FGF8*, XM_038579231.1; *Oryctolagus cuniculus FGF8*, XP_051679488.1; *Oxyura jamaicensis* *FGF8*, XP_035186264.1; *Calypte anna FGF8*, XM_008490410.2; *Columba livia* *FGF8*, XP_005507056.1; *Alligator mississippiensis FGF8*, XP_059585424.1; *Gopherus evgoodei* *FGF8*, XP_030426851.1; *Xenopus laevis FGF8*, BC169491.1; *Pseudophryne corroboree FGF8*, XP_063819806.1; *Cyprinus carpio FGF8a*, XP_042592960.1; *Ctenopharyngodon idella FGF8a*, XM_051918202.1; *Danio rerio FGF8a*, AAB82614.1; *Cyprinus carpio FGF8b*, XP_018964105.1; *Ctenopharyngodon idella* *FGF8b*, XM_051894416.1; *Danio rerio FGF8b*, AAI63362.1. The Ensembl accession numbers of *FGF8* used are as follows: *Larimichthys crocea FGF8a*, ENSLCRG00005018897; *Dicentrarchus labrax FGF8a*, ENSDLAG00005005948; *Larimichthys crocea FGF8b*, ENSLCRG00005003796; *Dicentrarchus labrax FGF8b*, ENSDLAG00005034123.

**
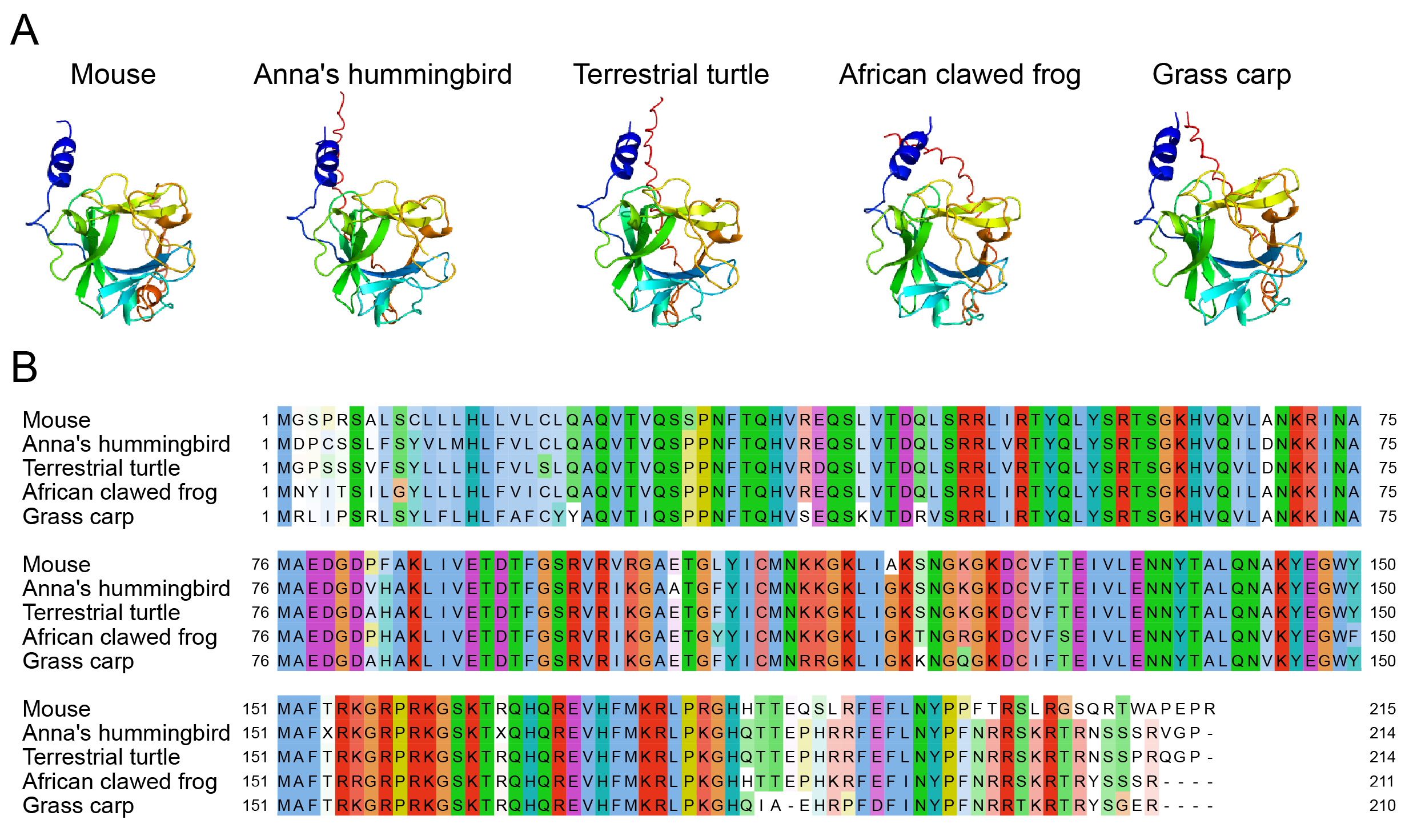
**

## Supplemental Fig. 2 Structure and sequence analysis of FGF8s in representative vertebrates. (A) The structural prediction of FGF8 proteins was performed using AlphaFold 2.0 software. (B) Coding sequence alignment of FGF8 in representative vertebrates. The multiple sequence alignment was conducted using the Clustal Omega program (https://www.ebi.ac.uk/jdispatcher/msa/clustalo) and then visualized by the Jalview program.


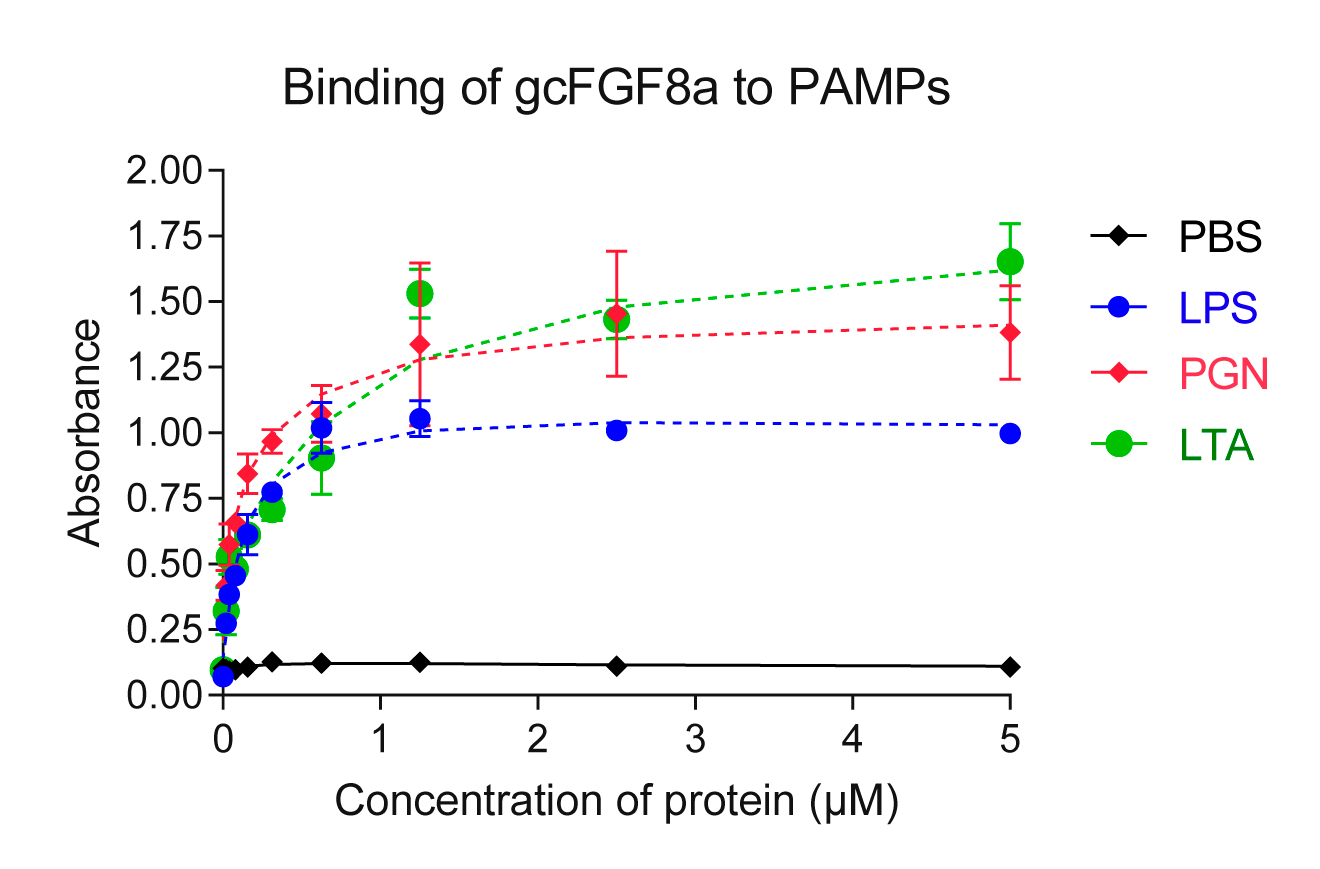


**Supplemental Fig. 3 Binding activity of gcFGF8a to PAMPs.** ELISA was employed to measure the binding activity of gcFGF8a to PAMPs, including LPS, PGN and LTA.


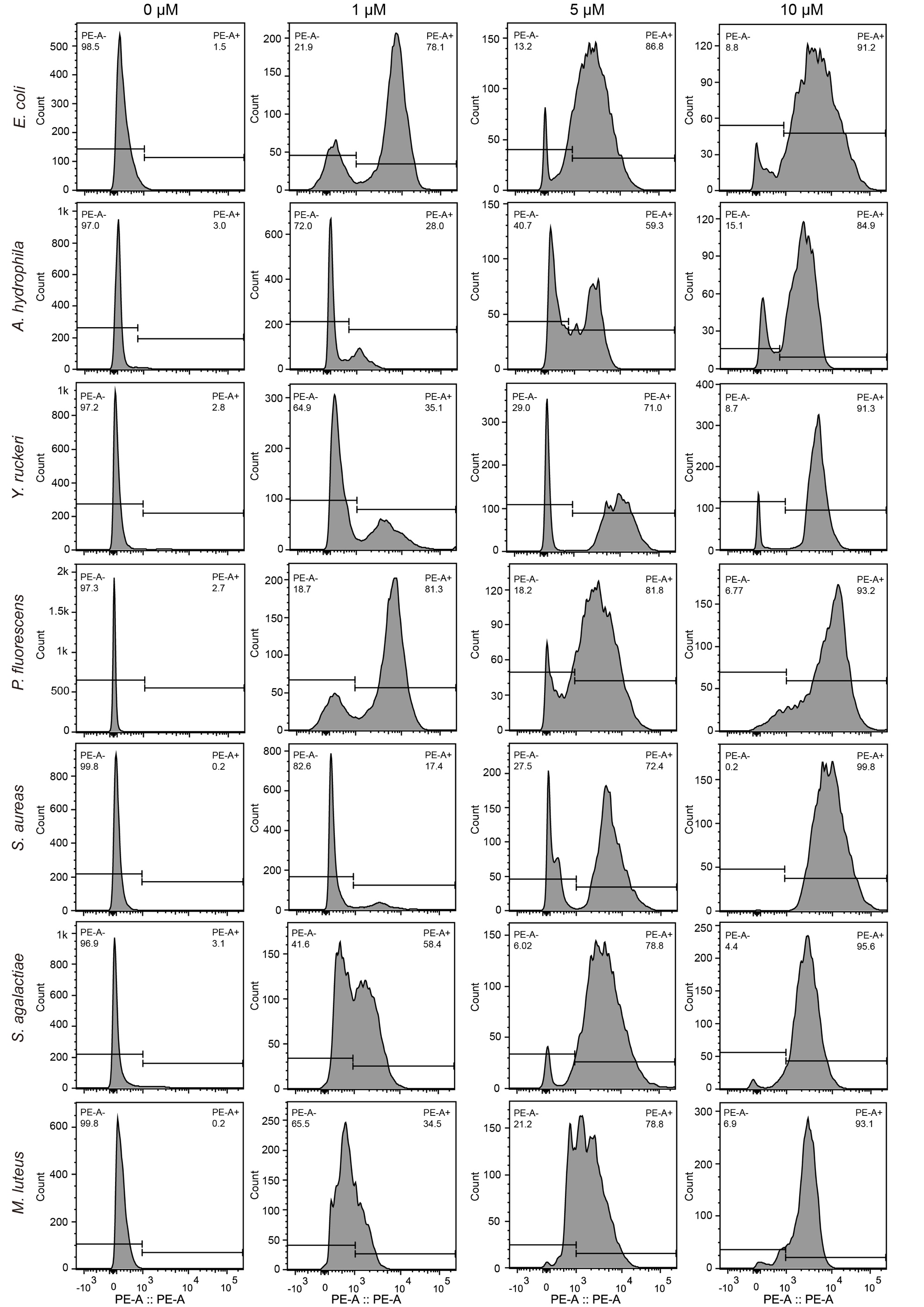


## Supplemental Fig. 4 Membrane permeability of bacteria treated with various concentrations of gcFGF8a. After treatment with different concentrations of gcFGF8a for 1 h, PI uptake by bacteria was measured using flow cytometry.


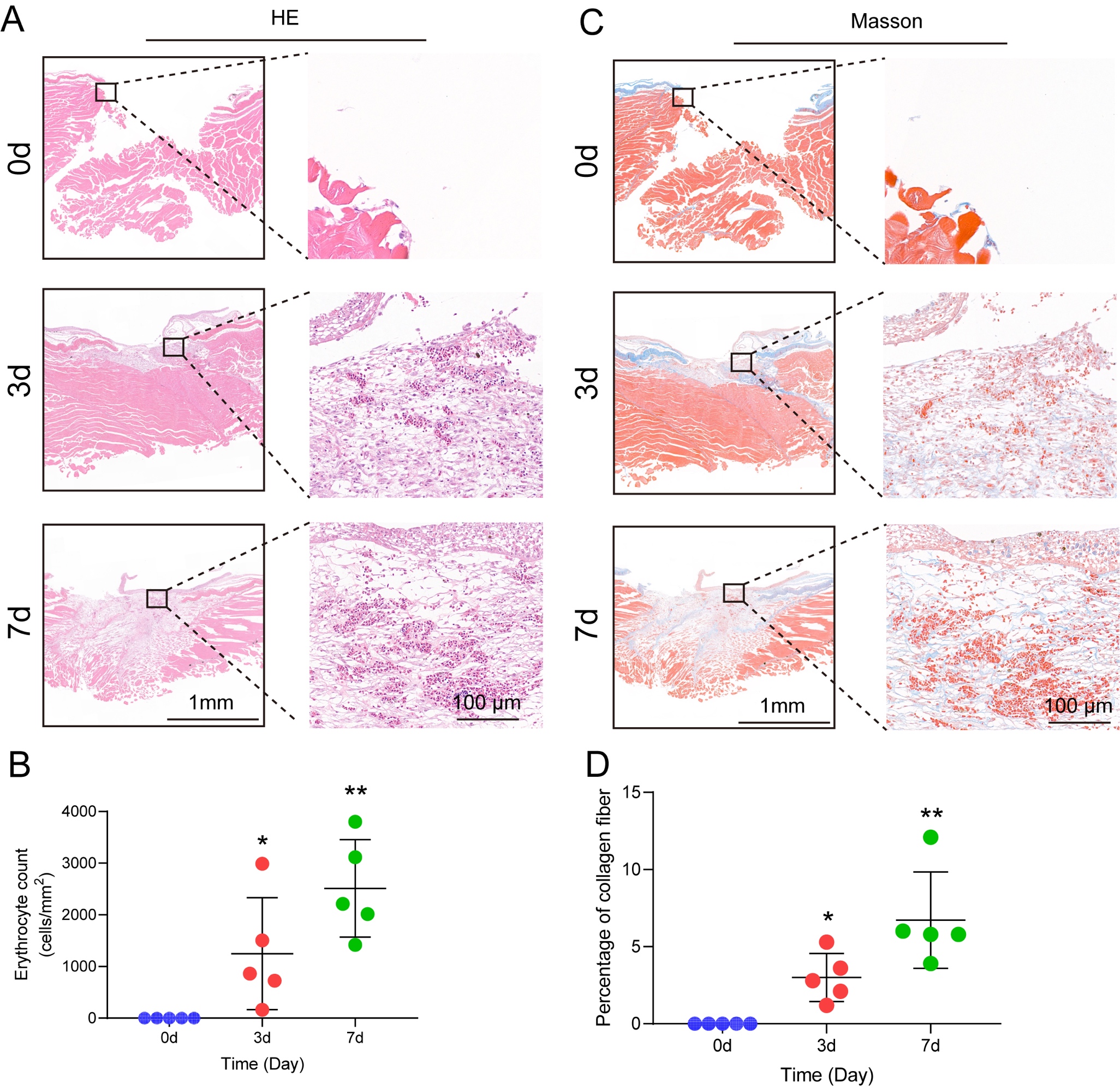


## Supplemental Fig. 5 Temporal progression of tissue damage, erythrocyte infiltration, and collagen deposition during wound healing. (A) HE staining of wound samples. The wound tissues were fixed, sectioned, and stained with HE at 0, 3, and 7 days after trauma to examine the tissue damage. (B) Quantification of erythrocyte content in the wounds. Erythrocytes in the wounds were counted from 5 randomly selected areas from slices. (C) Masson's trichrome staining of wound samples. The wound tissue was fixed, sectioned, and stained with Masson's trichrome at 0, 3, and 7 days after trauma to assess collagen fiber accumulation. Collagen fibers were dyed blue. (D) Measurement of collagen fiber densities in the wounds using Image Pro Plus software. Collagen fiber densities in the wounds were counted from 5 randomly selected areas from slices.


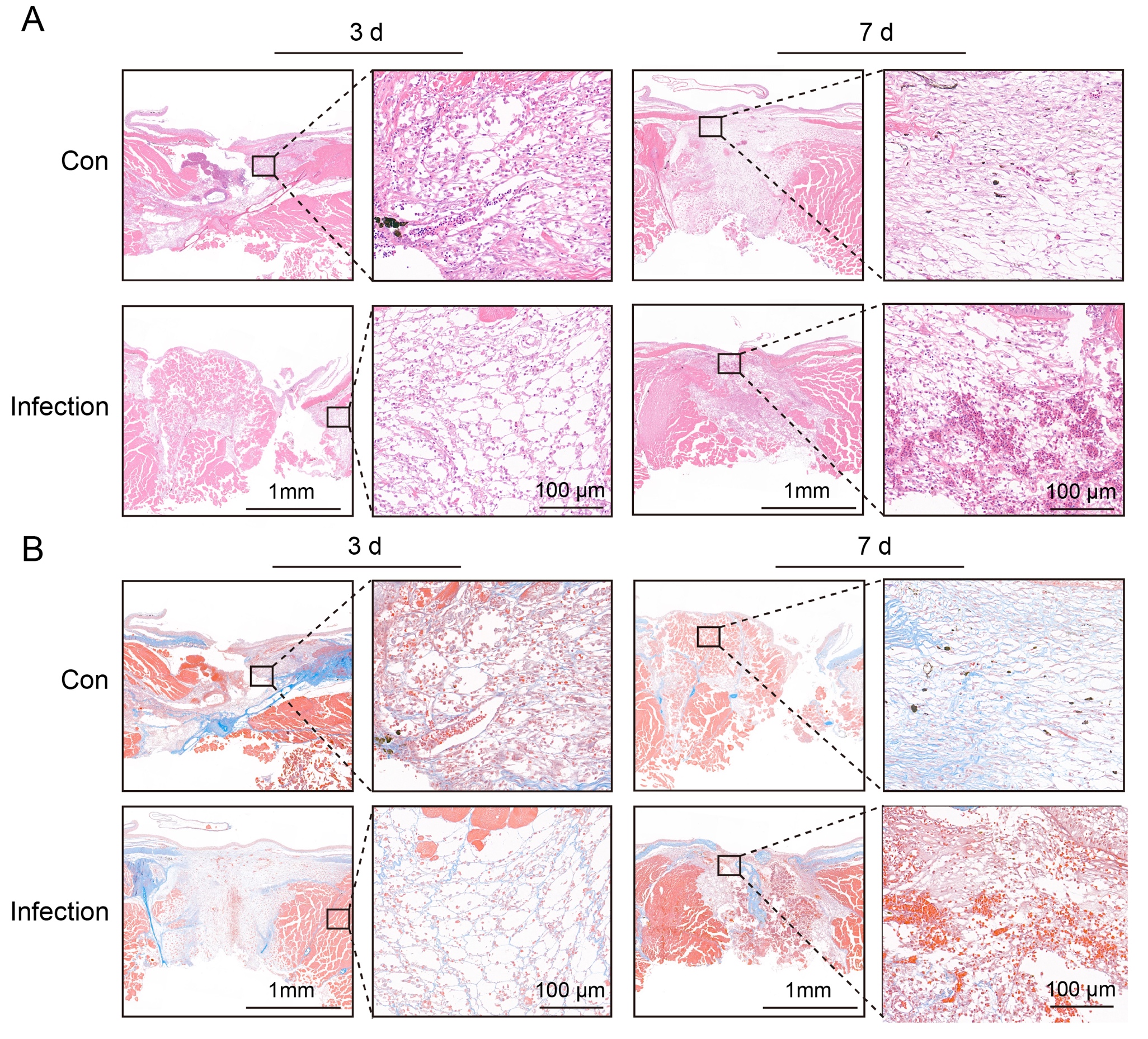


## Supplemental Fig. 6 *A. hydrophila* infection delays skin wound healing. (A) HE staining of wound samples. After bacterial infection, the wound tissues were fixed, sectioned and stained with HE at 0, 3 and 7 days after trauma to examine the tissue damage. (B) Masson's trichrome staining of wound samples. Wound tissue was fixed, sectioned, and stained with Masson's trichrome at 0, 3, and 7 days after trauma to assess collagen fiber accumulation.


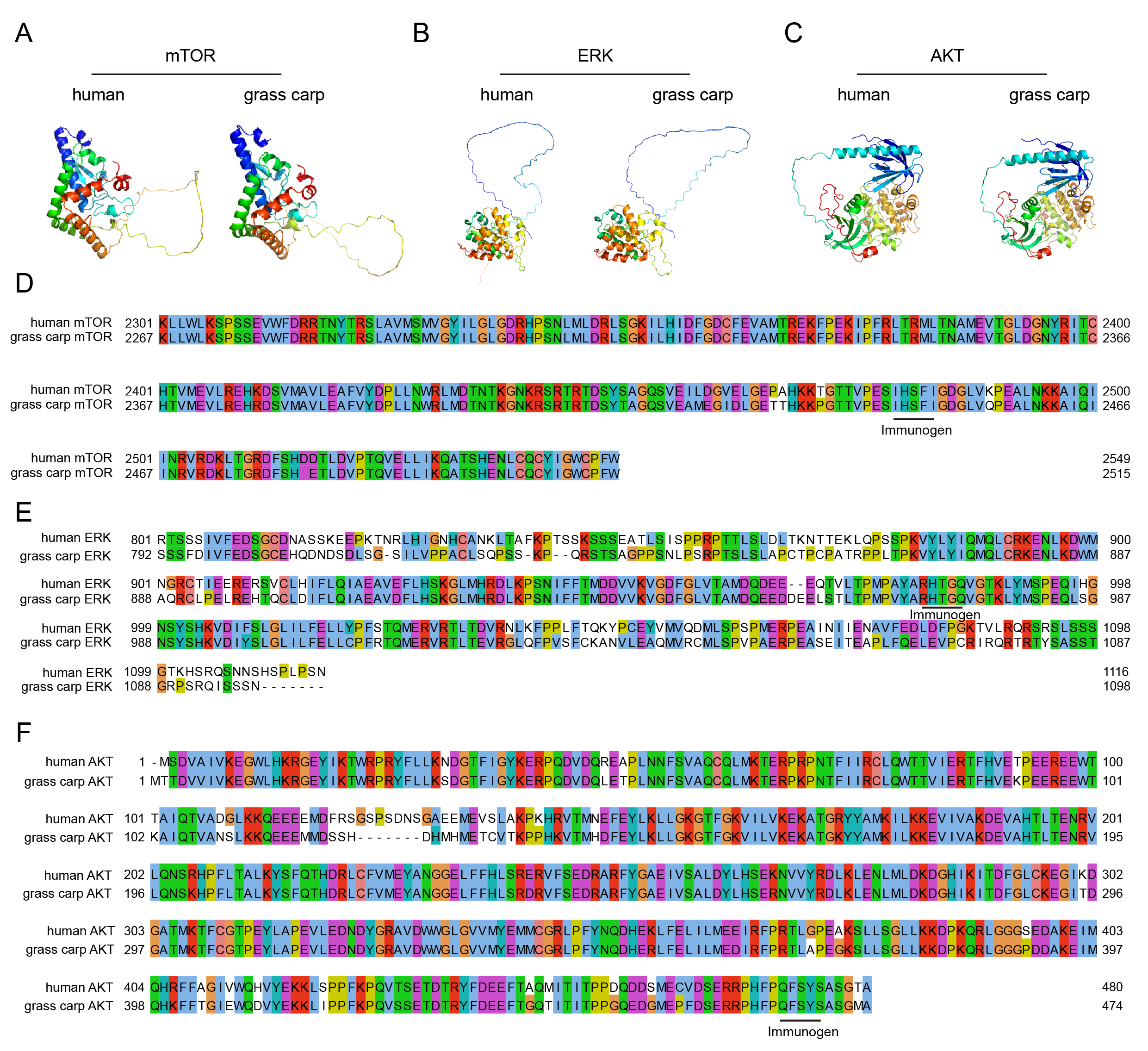


## Supplemental Fig. 7 Structural comparison and pairwise sequence alignment of mTOR, ERK, and AKT from human and grass carp. (A-C) The structural prediction of mTOR, ERK and AKT proteins was performed using AlphaFold 2.0 software. (D-F) Protein sequence alignment of mTOR (D), ERK (E), and AKT (F) from human and grass carp. The multiple sequence alignment was conducted using the Clustal Omega program (https://www.ebi.ac.uk/jdispatcher/msa/clustalo) and then visualized by the Jalview program.


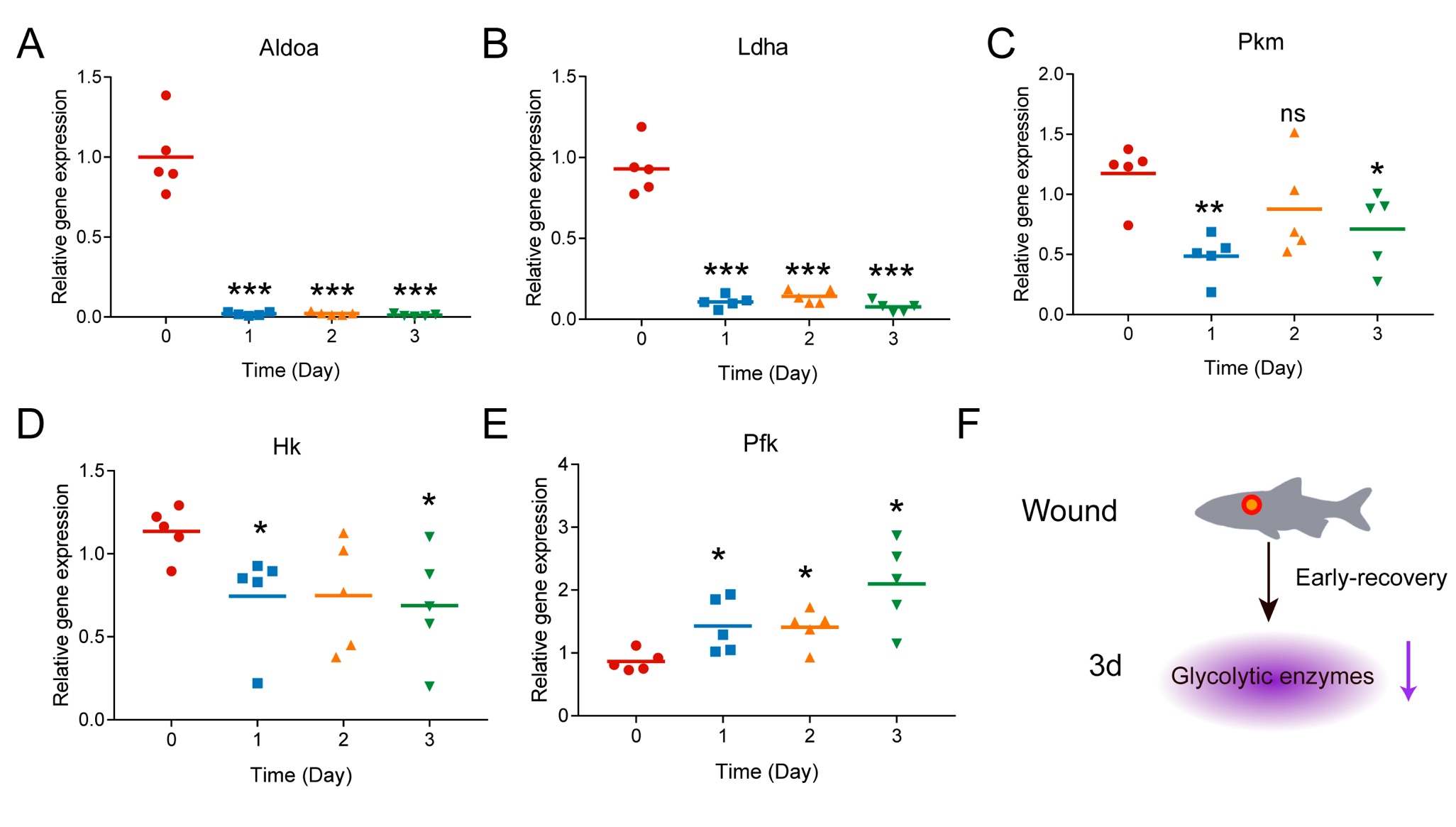


**Supplemental Fig. 8 Dynamic expression of glycolysis-related genes in wounds.** (A-E) Glycolysis-related genes are downregulated after trauma, except for *Pfk*. The fold changes of *Aldoa*, *Ldha*, *Pkm*, *Hk*, and *Pfk* were detected by RT-qPCR, using the 18S rRNA gene as the internal control, n = 5. (F) A predictive diagram illustrating the relationship between wound and glycolysis.


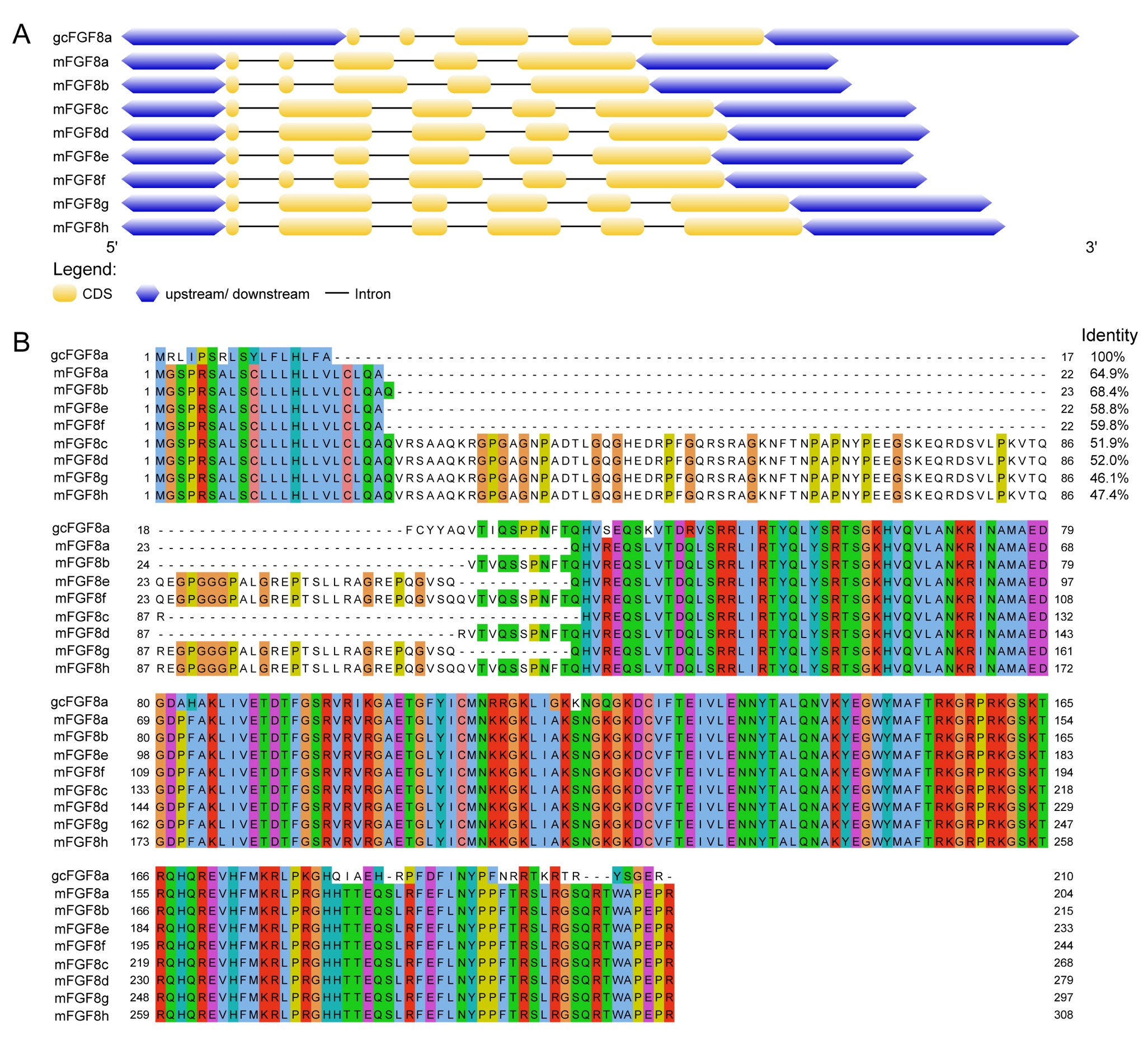


## Supplemental Fig. 9 Gene structure and multiple sequence comparison of gcFGF8a and mFGF8 splice variants. (A) Gene structure analysis of gcFGF8a and mFGF8 splice variants. (B) Protein sequence alignment of gcFGF8a and mFGF8 splice variants. The multiple sequence alignment was conducted using the Clustal Omega program (https://www.ebi.ac.uk/jdispatcher/msa/clustalo) and then visualized by the Jalview program.
